# Attenuate host susceptibility to respiratory virus invasion by inhibiting interactions between host proteins SLC16A3 and AP1G1

**DOI:** 10.1128/spectrum.03116-24

**Published:** 2025-09-08

**Authors:** Xinqi Deng, Rongge He, Jingsheng Zhang, Qingling Rao, Heng Chen, Zhixing Huang, ZiYi Hu, Shan Cao, Ziyu Wang, Shanshan Guo, Chunguo Wang, Xiaolan Cui

**Affiliations:** 1 Institute of Chinese Materia Medica, China Academy of Chinese Medical Sciences 71046, Beijing, China; 2 School of Chinese Materia Medica, Beijing University of Chinese Medicine 47839, Beijing, China; 3 Guizhou University of Chinese Medicine, Guizhou, China; 4 Beijing Research Institute of Chinese Medicine, Beijing University of Traditional Chinese Medicine 47839, Beijing, China; Barnard College, Columbia University, New York, New York, USA

**Keywords:** virus, host susceptibility, lactate, SLC16A3, AP1G1

## Abstract

Modulating cell endocytosis activity to reduce host susceptibility to virus represents a promising strategy for antiviral drug development. In this study, we reveal that lactate transporter SLC16A3 is a critical host factor for reducing diverse virus invasion. By performing metabolomics, proteomics, and thermal proteome profiling experiments, AP1G1, a pivotal protein involved in cellular endocytosis, was indiscriminately screened as a chaperone of SLC16A3. SLC16A3 decides the membrane enrichment of AP1G1 by protein interaction, thereby influencing host susceptibility to diverse viruses. This conclusion was further validated in SLC16A3 knocked-down cells, which indicated a broad-spectrum target for anti-virus drug development to recede virus entry by blocking the interaction between AP1G1 and SLC16A3. This conclusion has been validated with a patent medicine Shufengjiedu (SFJD). SFJD exhibits strong effect in decreasing the susceptibility of host cells to viral infections. Molecularly, SFJD administration results in disrupting the interaction between SLC16A3 and AP1G1 and reduced membrane localization of AP1G1. Consequently, it achieves a receded endocytosis activity of host cells on viral particles. This provides evidence for the practicability of the SLC16A3-AP1G1 strategy. Taken together, the regulation on the SLC16A3-AP1G1 interaction represents a broad-spectrum and practicable antiviral strategy. This study offers insights into a novel approach for inhibiting viral infections through the alteration of host susceptibility and advances the idea for antiviral drug development.

**IMPORTANCE:**

We have discovered that a broad-spectrum antiviral strategy, highlighting the lactate transporter SLC16A3 as a critical determinant of host cell susceptibility to viruses. SLC16A3 was found to interact with AP1G1, which is a pivotal protein involved in cellular endocytosis. Disrupting the interaction between AP1G1 and SLC16A3 leads to reduced membrane localization of AP1G1, thereby reducing the host cell endocytosis of viral particles. Importantly, we found that the patent medicine Shufengjiedu (SFJD can significantly reduce the susceptibility of host cells to viral infection through this mechanism, providing evidence for the practicability of SLC16A3-AP1G1 strategy. Taken together, the modulation on the SLC16A3-AP1G1 interaction represents a broad-spectrum and practicable antiviral mechanism. This study offers novel insights into strategies for inhibiting viral infections through the alteration of host susceptibility and advances the idea for antiviral drug development.

## INTRODUCTION

Respiratory viral infections are an important public health issue globally. Respiratory viral infections are caused by the invasion of respiratory viruses. The most common respiratory viruses include respiratory syncytial viruses, influenza viruses (Influenza AH1N1, Influenza H1N1), and coronaviruses (HCoV-229E, SARS-CoV-2) (1) Worldwide, approximately 290,000 to 650,000 people die from influenza-related respiratory viral infections each year (2, 3). The receptors for invasion of host cells by different respiratory viruses are specific. Respiratory syncytial virus mainly binds to G proteins on the surface of host cells (4), influenza virus binds to the sialic acid receptor of host cells through its surface hemagglutinin (5), and coronaviruses (e.g., SARS-CoV-2) rely mainly on their spike proteins to bind to the ACE2 receptor of host cells (6). The rapid mutation of viruses after invasion makes it difficult to keep vaccine updates up with mutation. Additionally, the use of vaccines alone against viruses has certain limitations and drawbacks (7–9). On the other hand, cell endocytosis activity after receptor binding determines virus invasion efficiency (10). Thus, a general target to recede host cell endocytosis would provide greater application potentials for resisting diverse viruses.

Lactate metabolism is a highly active process in host cells during viral infections. The concentration of lactate indicates the severity of the viral infection and the condition of the host cell, which can help evaluate the prognosis for patients with respiratory viral infections (11, 12). Besides, enveloped viruses’ fusion to the host cell membrane was detected more efficiently at an acidic pH. Thus, it is supposed that increased extracellular acidification resulting from higher lactate exocellular abundance may benefit the virus endocytotic pathway and promote virus infection (13–15). Though the 2-deoxy-D-glucose (2-DG), which is a reagent that inhibits lactate production, has exhibited its antiviral activity in diverse infections (16, 17), it is attributed to the interference on the virus gene expression and replication (18, 19), rather than host endocytosis on virus. In addition, as a metabolic modifying drug, 2-DG was considered to be used with caution because of its side effect (20). Therefore, it remains to be discussed whether the lactate level is the direct factor that is involved in the endocytosis activity of host cells, and it may be worthier to discover new strategies for antivirus from lactate transporter activity.

In this study, we revealed that lactate transporter SLC16A3 is important for host cell susceptibility to viruses since it interacts with endocytosis factor AP1G1 and decides the performance of virus endocytosis. Thus, SLC16A3-AP1G1 interaction is a potential target for antiviral treatment by lowering host susceptibility to viruses. Further, a patent medicine Shufengjiedu (SFJD) was found to exert antiviral effect via disrupting the interaction between SLC16A3 and AP1G1, which provides evidence for the practicability of SLC16A3-AP1G1 strategy. Collectively, we explore a broad-spectrum antiviral mechanism by regulating host susceptibility to viruses.

## MATERIALS AND METHODS

### Chemicals and reagents

Methanol, formic acid, and acetonitrile were purchased from ThermoFisher Scientific. Trypsin (modified, sequencing grade), urea (Promega), A/G beads, 2-deoxy-D-glucose (2-DG) were purchased from MedChemExpress (Shanghai, China). CCK-8 assay kit was purchased from Beyotime Biotechnology (Shanghai, China). Native Lysis Buffer, BCA Protein Assay Kit was Solarbio Science & Technology Co., Ltd. (Beijing, China). Primary antibodies of AP1G1 (NBP3-15727) were purchased from Novus Biologicals (Shanghai, China), and SLC16A2 (20676-1-AP) was obtained from Proteintech (Wuhan, China). SLC16A3 (ab74109) and SLC16A7 (ab129290) were purchased from Abcam (Cambridge, UK). The other chemicals were purchased from Sigma–Aldrich unless stated otherwise.

Patent medicine SFJD (Batch number: 3201020) was obtained from Anhui Jiren Pharmaceutical Co., Ltd. (Anhui, China) with a quality control file provided in supplementary material (Patented drug quality inspection report).

### Animal

Male-specific pathogen-free Kunming (KM) mice (20-30g) were provided by Vital River (Beijing) Laboratory Animal Technology Co., LTD, with animal license number SYXK (Beijing) 2020-0042. They were housed in the standard barrier environment of the Institute of Traditional Chinese Medicine of China Academy of Chinese Medical Sciences, with free food and a 12-h light/dark cycle. Before the experiment, the mice were given unrestricted standard chow and clean drinking water. Animal experiments were performed by the requirements of international ethics for laboratory animals, and the ethics number is ICMM-2022D052.

Besides the control group, the mice in the other groups were infected by nasal drip with 100 TCID50 influenza A virus (H1N1) /2009 TC isolate, human respiratory syncytial virus (RSV, strain 18537), or human coronavirus 229E (strain 229E), with an administration volume of 40 µL for each infection. The drug was administered i.g. daily for 5 days starting on the day of infection. The dose of SFJD administered to mice was 0.572 g/kg once daily. The control and model groups were given distilled water. On day 5 of administration, blood and lung tissues were harvested from mice. Then, 500 mg/kg 2-DG was applied to mice.

### Cell cultures and virus

Human Cell BEAS2B was purchased from BeNa culture collection (Beijing, China) and cultured in Dulbecco’s Modified Eagle’s Medium (DMEM, Pricella, China) supplanted with 10% fetal bovine serum (FBS, HyClone, United States) and 1% penicillin-streptomycin (Pricella, China) at 37°C under 5% CO_2_ atmosphere.

The viral stocks of H1N1, RSV, and 229E were kept and operated according to the SOP of Centers for Biosafety of China Academy of Chinese Medical Sciences (Beijing, China). Experiments with H1N1, RSV, and 229E were carried out in biosafety level 2 (BSL2) containment in compliance with institutional guidelines

### Cell viability assay

Cell viability was used to measure the proliferation rates of cells. Here, 100 μL of treated cells were inoculated and cultured in 96-well plates for 24 h, and the proliferation rate of cells was detected using the Cell Counting Kit (CCK-8, Solarbio, China). The OD value was recorded at 450 nm by a microplate spectrophotometer (KeRuiEnTe Technology Co. LTD, Beijing, China).

### Protein sample preparation

Lung tissue (six biological replications of each group) lysates were collected and stored in aliquots at −20°C after centrifugation at 16,000×*g* for 30 min at 4°C after homogenization and lysis. For high-throughput proteomics, the samples were digested with trypsin and processed with the FASP method which was described by Wisniewski et al (21). For Western blotting, the samples were heated at 100°C with loading buffer for 5 min.

### Western blotting and quantitative polymerase chain reaction (qPCR) assay

Concentrations of protein samples were measured using BCA protein assay kit (Solarbio, China) and adjusted to 1 µg/µL in loading buffer. Equivalent amounts of protein were loaded per lane and resolved by 12% SDS-PAGE. Protein was transferred to PVDF membrane (Millipore) and blocked with 5% nonfat milk in TBST overnight at 4°C. Membranes were cut or using Western blot antibody stripping solution to allow targeting of multiple size proteins on the same blot. Membranes were incubated with diluted primary antibody for 2 h at room temperature with shaking. Images were developed by a chemiluminescence imager after horseradish peroxidase labeled secondary antibodies incubation.

TRIzol (Invitrogen) and TIANScript RT Kit (TIANGEN) were applied for RNA extraction and reverse transcription following the manufacturer’s instructions. Then, 1 µg of total RNA was applied in cDNA synthesis with random primers and M-MLV (H-) reverse transcriptase (Vazyme). The PCR kits used for the three viruses were purchased from Shanghai ZJ Bio-tech Co., Ltd, with the following catalog numbers: RR-0051-02 for H1N1, RR-0098-02 for RSV, and RR-0197-02 for 229E. qPCR was performed in triplicate on Applied Biosystems 7500 Real-Time PCR System (Thermo Fisher). Data were calculated with the formula for relative FC = 2‐ΔΔCT.

### Immunoprecipitation—Western blot assay

BEAS2B cells were grown to 80% confluence and transfected with GST-AP1G1 and FLAG-SLC16A3 plasmids to express the tagged target proteins. Following transfection, the cells were washed three times with PBS and lysed by sonication on ice. Cell lysates were collected by centrifugation at 20,000×*g* for 30 min at 4°C to remove debris. The protein concentration was determined using the BCA protein assay kit.

For immunoprecipitation (IP), GST antibodies were incubated with magnetic beads at 4°C for binding. The beads were washed three times with PBS containing 0.5% Tween 20, followed by three additional washes with PBS (low-speed centrifugation at 3,000 rpm for 2 min each). Sample protein lysate was added to the beads and rotated overnight at 4°C to allow binding. Afterward, the beads were washed again: three times with PBS containing 0.5% Tween 20, followed by three washes with PBS (low-speed centrifugation at 3,000 rpm for 2 min each). The bound proteins were eluted to obtain the bait protein and its interacting proteins.

Finally, the interactions between SLC16A3 and AP1G1 were detected using FLAG and GST antibodies by Western blotting.

### Cloning and recombinant protein expression and purification

AP1G1 and SLC16A3 sequences were cloned into N-Sumo-affinity tag containing vector pSumo- mut (Novagen). The protein was expressed in Escherichia coli BL21 (DE3) cells. Cells were grown in LB media supplemented with 200 µg/mL kanamycin at 37°C to OD600 of 0.6–0.8. Overexpression was induced with 0.2 mM IPTG at 37°C overnight. Cells were harvested by centrifugation and lysed by ultrasonication. The cell lysates were clarified by centrifugation, and the soluble fractions were purified by Ni-IDA resin (Novagen). Recombinant protein was then loaded to a dialysis bag overnight for further purification

### Quantitative proteomics

LC-MS/MS data were collected using an Easy-Nalc1200 system (Thermo Fisher Scientific) combined with an Orbitrap Fusion Lumos mass spectrometer (Thermo Fisher Scientific, Bremen, Germany). Peptides were separated by using PepMap C18 75 µm × 250 mm 2 µm columns (Dr. Maisch GmbH, Germany). Mobile phase A was 0.1% formic acid aqueous solution, mobile phase B was 80% acetonitrile solution, gradient elution: 0–2 min, 2%–8% B; 2–112 min, 8%–32% B; 112–114 min, 32%–95% B; 114–120 min, 95% B. Data-dependent scanning was used for mass spectrometry and the top-20 method for MS/MS scanning. Scanning range: m/z 300-1800; MS resolution: 70,000; MS/MS cracking mode: HCD; cracking energy: 30; resolution: 17,500; AGC: 5 × 106; spray voltage: 2.1 kV; temperature of ion transmission tube: 250°C. XCalibur (version 4.2; Thermo Fisher Scientific) was used for data processing.

### Bioinformatics KEGG protocol

First, a list of proteins was prepared in a format compatible with the KEGG database. The data were then uploaded to the KEGG database (version 2023-06), accessible at https://www.genome.jp/kegg/, where we performed the enrichment analysis to visualize relevant pathways. We established a significance threshold with a *P*-value of less than 0.05. The results were subsequently exported for further visualization and analysis using GraphPad Prism.

### Immunofluorescence staining

Cells were transfected with plasmids containing GST-tagged AP1G1 (vector: pcDNA3.1-N-GST-TEV) and FLAG-tagged SLC16A3 (vector: pcDNA3.1-3xFlag) sequences to produce the tagged proteins. These plasmids were obtained from Changsha ZQ Biotechnology Co., Ltd.

After transfection, cells were fixed with 4% paraformaldehyde for 15 min at room temperature to preserve cellular morphology. Following fixation, the cells were washed three times with PBS to remove excess fixative.

To permeabilize the cells, we incubated them with 0.5% (v/v) Triton X-100 in PBS for 10 min, allowing for antibody penetration. After permeabilization, the cells were blocked with 3% (w/v) BSA in PBS for 1 h at room temperature to minimize non-specific binding.

Subsequently, the cells were incubated overnight at 4°C with primary antibodies recognizing the respective tags (1:200), followed by incubation with fluorescent secondary antibodies. The cells were then incubated with the corresponding secondary antibodies for 2 h at room temperature, protected from light to prevent degradation of the fluorescence. After this incubation, the cells were washed again with PBS.

Finally, images were captured using a confocal laser scanning microscope (Leica SP8) and processed using LAS X software (Leica).

### Subcellular fractionation

Cells were washed and lifted with PBS containing 2.5 mM EDTA. Then, they were washed in 1 mL of hypotonic buffer (10 mM Tris pH 7.4, 1.5 mM MgCl2, 10 mM KCl with protease inhibitors). The cells were centrifuged (400×*g* for 3 min at 4°C) and resuspended in 750 µL of hypotonic buffer, followed by incubation on ice for 30 min. Swelled cells were homogenized by passing them through a 26-gage needle. Nuclei and cell debris were precipitated by centrifugation at 2,500×*g* and 4°C for 5 min. A portion of the post-nuclear supernatant was used as input control for Western blotting. The supernatants were centrifuged at 17,000×*g* and 4°C for 30 min to pellet the cellular membranes. The supernatant was discarded, and the membrane pellet was washed thrice with hypotonic buffer. Immunoblotting samples were prepared by adding 5× SDS loading buffer to the cytosolic fraction and resuspending the membrane pellet in an equal volume of 1× SDS loading buffer. The samples were heated to 95°C for 10 min and analyzed by wWestern blotting. Tubulin served as control markers for the cytosolic fraction.

### sh-RNA and lentivirus infection

shRNA lentivirus was prepared by the co-transfection of 293T cells with pLKO.1 and packaging plasmids pMD2G and psPAX2. Then, 48 h after transfection, the viral supernatants were collected. The BEAS2B cells were incubated with viral supernatants in the presence of polybrene. The positive cells were selected with 1 µg/mL puromycin. The target sequences were AP1G1 shRNA-1: 5′- GGAATAATATCCGAGGCATGA-3′, AP1G1 shRNA-2:5-GCATTGTCCCAGCATTTAACA-3′, SLC16A3 shRNA-1: 5′- GGAACAAGCCACTTTATTCAC-3′, SLC16A3 sh RNA-2: 5′- GCCACTTTATTCAC TGCTGTG-3′. Scramble sequence was used as negative controls. The Cys→Ala mutation was introduced by site-directed mutagenesis into the AP1G1 cDNA and cloned into the plasmid pGEX-4T-1.

### Enzyme-linked immunosorbent assay (ELISA)

The levels of TNF-α, IL-1, and IL-6 in lung tissues were measured using commercially available ELISA kits from Enzyme-linked Biotechnology Co., Ltd. (Shanghai, China).

## RESULTS

### Lower lactate expression levels cannot reduce viral load of host

**Fig 1:**
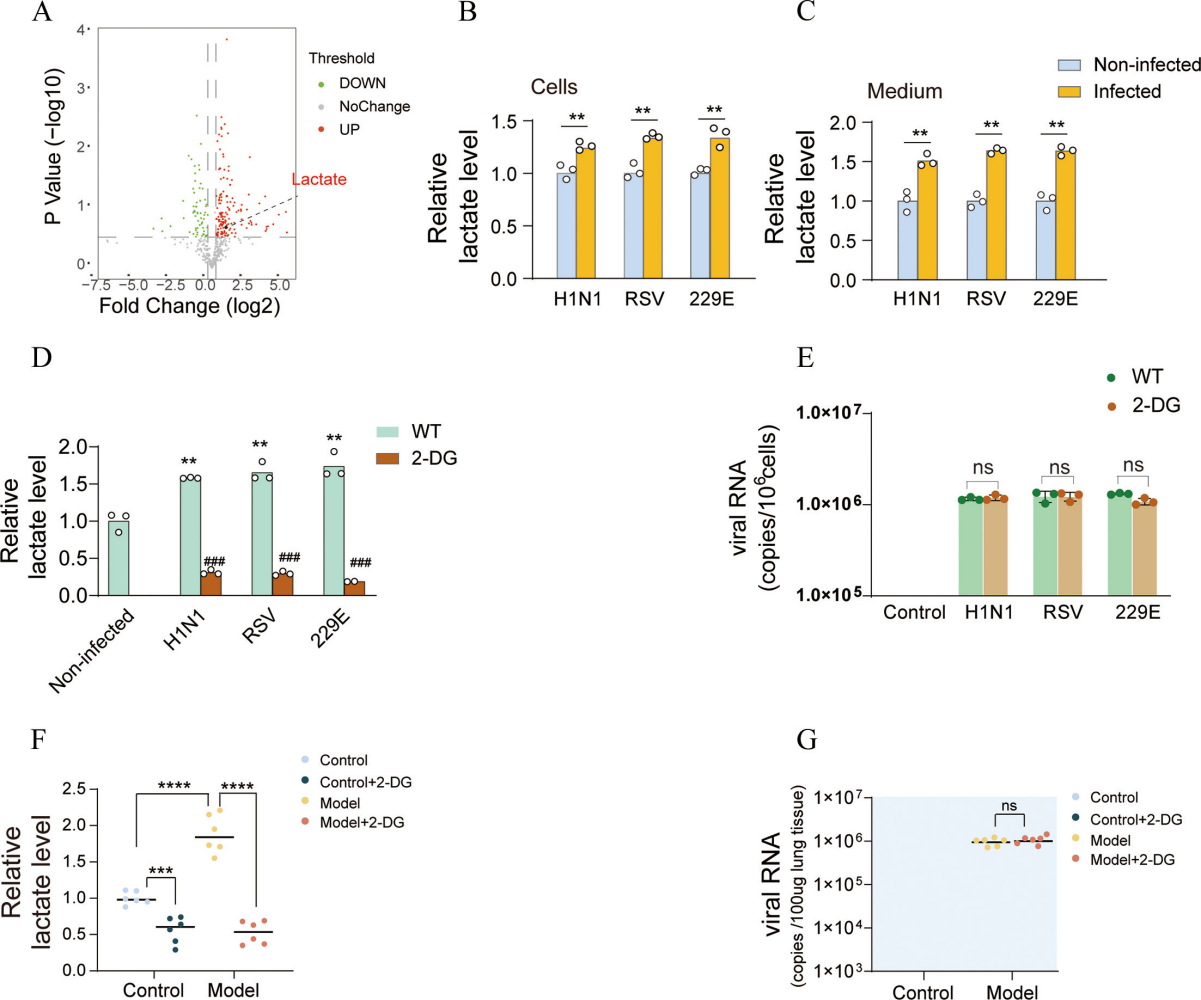
Lactate level and viral genome copies. (**A**) The volcano plot of the relative abundances of metabolites of control and model group. Mice in the model group were subjected to H1N1 (100 TCID50) for 5 days. (**B, C**) Relative itaconate level in BEAS2B cell body and culture medium following H1N1, RSV, and 229E virus infection. *t*-test, **P < 0.01. (**D–E**) Lactate level and viral genome copies after 2-DG (20 mM) administration in BEAS2B cells. Cells were infected with H1N1, RSV, 229E (MOI = 2). *t*-test, ***P < 0.001, ****P < 0.0001, ^###^*P* < 0.001. (**F, G**) Lactate level and viral genome copies of animals (H1N1 dosage = 100 TCID50, 2-DG dosage = 500 mg/kg). *t*-test, ***P < 0.001, ****P < 0.0001, *n* = 6.

Principal component analysis (PCA) on metabolomic data revealed distinguished differentiation caused by H1N1 infection (Fig. S1A and B). Notably, an elevation in lactate expression levels was found in the serum of mice infected with H1N1 (Fig. 1A). Furthermore, lactate concentrations were significantly increased in both BEAS2B cell bodies (Fig. 1B) and the culture medium (Fig. 1C) subsequent to the infections of H1N1, RSV, and 229E viruses. The administration of 2-DG caused a significant decrease in lactate levels in cells (Fig. 1D). However, it exerted little impact on the invasion of H1N1, RSV, and 229E (Fig. 1E). Likewise, the lung of mice infected with the H1N1 virus, 2-DG treatment led to a significant reduction in lactate expression levels (Fig. 1F), but no alteration in viral load (Fig. 1G). These findings indicate that lactate levels increase following viral infection of host cells and are associated with the status of viral infection. Nevertheless, the inhibition of lactate expression did not result in a lower viral load of host.

### SLC16A3 protein has influence on host cell susceptibility to viruses

**Fig 2:**
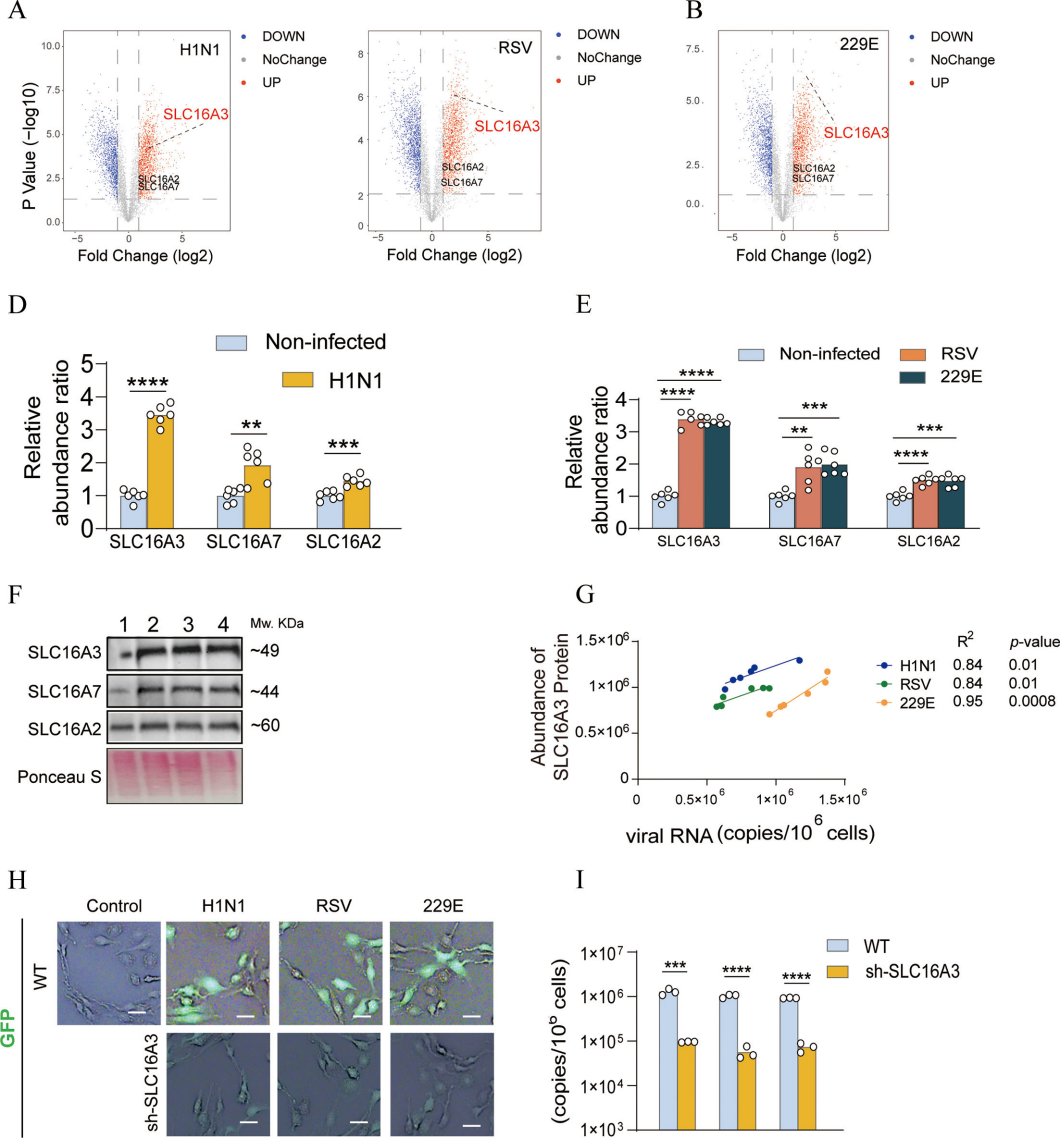
SLC16A3 influences the susceptibility of host cells to viral infections. (**A–C**) Proteomic data of lactate transporter proteins in response to H1N1, RSV, or 229E infection. (**D, E**) ELISA data of lactate transporter proteins in response to H1N1, RSV, or 229E infection. *t*-test, **P < 0.01, ***P < 0.001, ****P < 0.0001, *n* = 6. (**F**) Western blot illustrated the levels of lactate transporter proteins in response to H1N1, RSV, or 229E infection. (M, lane 1 = Non-infected, 2 = H1 N1, 3 = RSV, 4 = 229E.) The data represent three replicates. (**G**) SLC16A3 expression levels in cells upon different dosages of H1N1, RSV, or 229E infections. *t*-test, **P < 0.01, ***P < 0.001, ****P < 0.0001, *n* = 6. (**H**) BEAS2B cells were infected with GFP-tagged H1N1, RSV, and 229E viruses (MOI = 2) at 24 hpi. The GFP signal was attenuated in the sh-SLC16A3 group compared with the WT group, indicating reduced virus invasion. Scale bar  =  100 µm. The data represent three replicates. (**I**) Viral genome copies in BEAS2B cells. Cells were infected with H1N1, RSV, and 229E virus (MOI = 2). Cells were transfected with sh-SLC16A3 plasmid. *t-*test, ***P < 0.001, ****P < 0.0001, *n* = 3.

To further explore the association between lactate metabolism and viral infections, we carried out UPLC-HRMS-based proteomics to reveal significant alterations in proteins. Among the 1,117 differentiated proteins, 622 proteins exhibited an upregulation of >1.50 fold in the model group (relative to the control group), while 495 proteins demonstrated a downregulation of <0.666-fold. The expression levels of lactate transporter proteins, including SLC16A2, SLC6A7, and SLC16A3, were all altered in host cells upon viral infection of H1N1, RSV, or 229E (Fig. 2A through F). Among these proteins, SLC16A3 displayed the most significant alteration with >3-fold increase, which was notably greater than the changes in the other two proteins. Additionally, it displays a positive correlation between SLC16A3 expression levels and viral load (H1N1, R²=0.84; RSV, R²=0.84; 229E, R²=0.95) (Fig. 2G), which suggested a strong link between SLC16A3 protein and host susceptibility to viral infections. In the GFP-viral tracing assay, the attenuated green fluorescence in the sh-SLC16A3 cells demonstrated that virus invasions were reduced upon SLC16A3 afunction (Fig. 2H). Consistently, lower viral loads were found in the SLC16A3-knocked-down cells (Fig. 2I). In summary, the SLC16A3 protein functions as a pivotal factor in modulating the susceptibility of host cells to viral infections.

### Diminishing the interactions between SLC16A3 and AP1G1 leads to a decrease in the endocytic uptake of viruses by host cells

**Fig 3:**
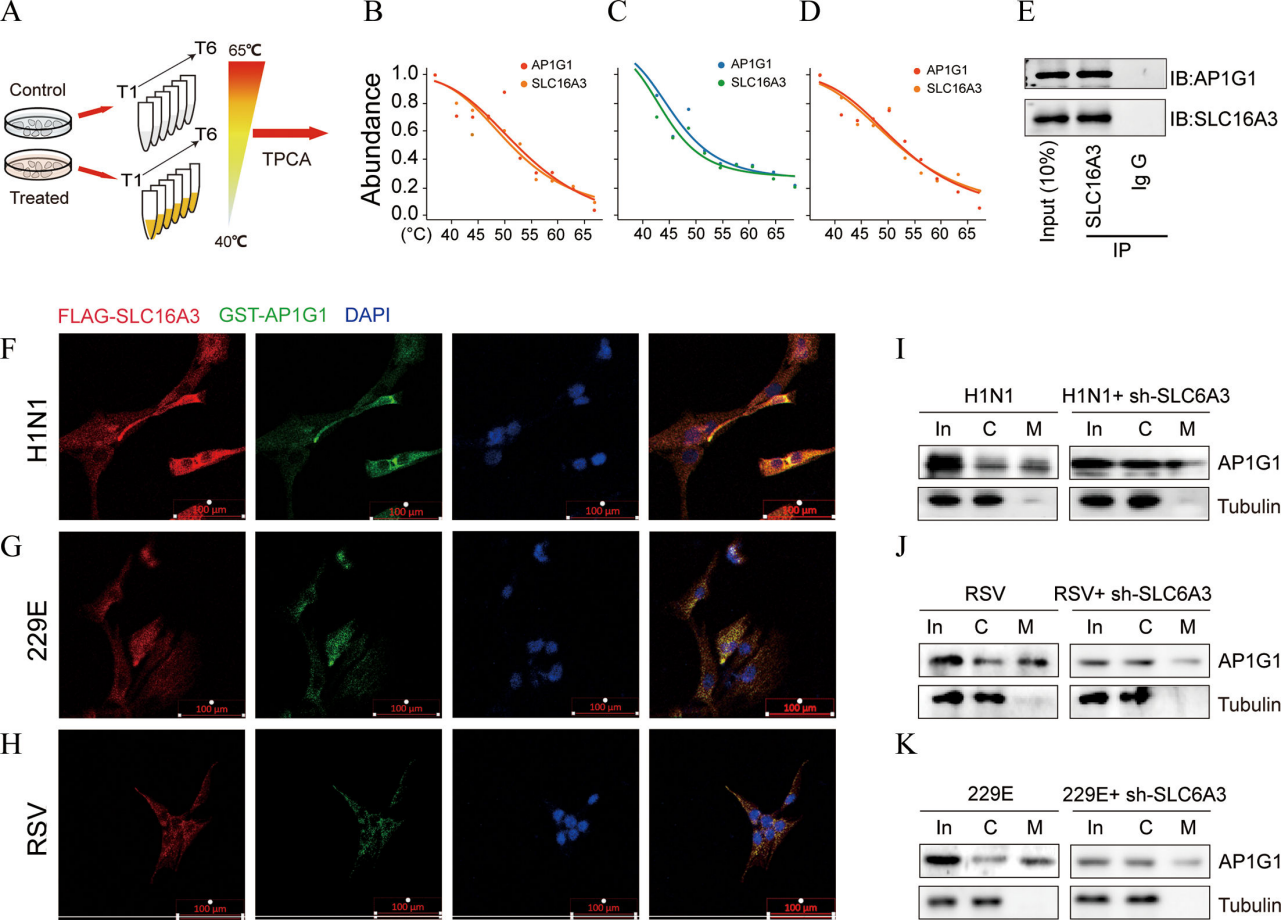
The interactions between SLC16A3 and AP1G1 (**A**) TPCA-MS experimental scheme. (**B–D**) SLC16A3 and AP1G1 exhibit similar melting curves. Cells were non-infected or infected with H1N1, RSV, and 229E viruses. (**E**) IP-WB result demonstrating the interaction between SLC16A3 and AP1G1. (**F–H**) Immunofluorescence images illustrating the subcellular localization of SLC16A3 and AP1G1. BEAS2B cells were infected with H1N1, RSV, and 229E viruses. (**I–K**) Western blot assay showing the subcellular localization of AP1G1. In = input, C = cytoplasm, M = cytomembrane. Cells were infected with H1N1, RSV, and 229E virus (MOI = 2, *n* = 3).

To further elucidate the mechanism by which SLC16A3 influences the susceptibility of host cells to viral infections, we investigated the proteins that interact with SLC16A3 during such infections. Thermal proximity coaggregation coupled MS (TPCA-MS) experiments (Fig. 3A) indicated that SLC16A3 and AP1G1 exhibited comparable melting curves (Fig. 3B–D), suggesting a potential interaction between these two proteins. Furthermore, immunoprecipitation followed by Western blotting (IP-WB) corroborated the interaction between SLC16A3 and AP1G1 (Fig. 3E). Cellular immunofluorescence staining (Fig. 3F–G) revealed co-localization of SLC16A3 and AP1G1 in BEAS2B cells, thereby reinforcing the evidence of their interaction. Subsequent analysis of the AP1G1 protein content following the isolation of the cell membrane from the cytoplasm demonstrated a significant reduction in the membrane enrichment of AP1G1 upon the knockdown of SLC16A3 (Fig. 3I through K; Fig. S2), underscoring the critical role of SLC16A3 in the membrane localization of AP1G1. AP1G1 has been recognized as a vital host factor in the context of viral infections (22), and its membrane enrichment is essential for the endocytic processes of host cells (23). Consequently, these findings imply that targeting the interaction between SLC16A3 and AP1G1 represents a universal strategy for modulating host susceptibility to viral infections. Inhibiting the SLC16A3-AP1G1 interaction could potentially diminish host cell vulnerability to a range of viruses, thereby offering significant insights for the development of antiviral therapeutics.

### SFJD chemical composition analysis

**Fig 4:**
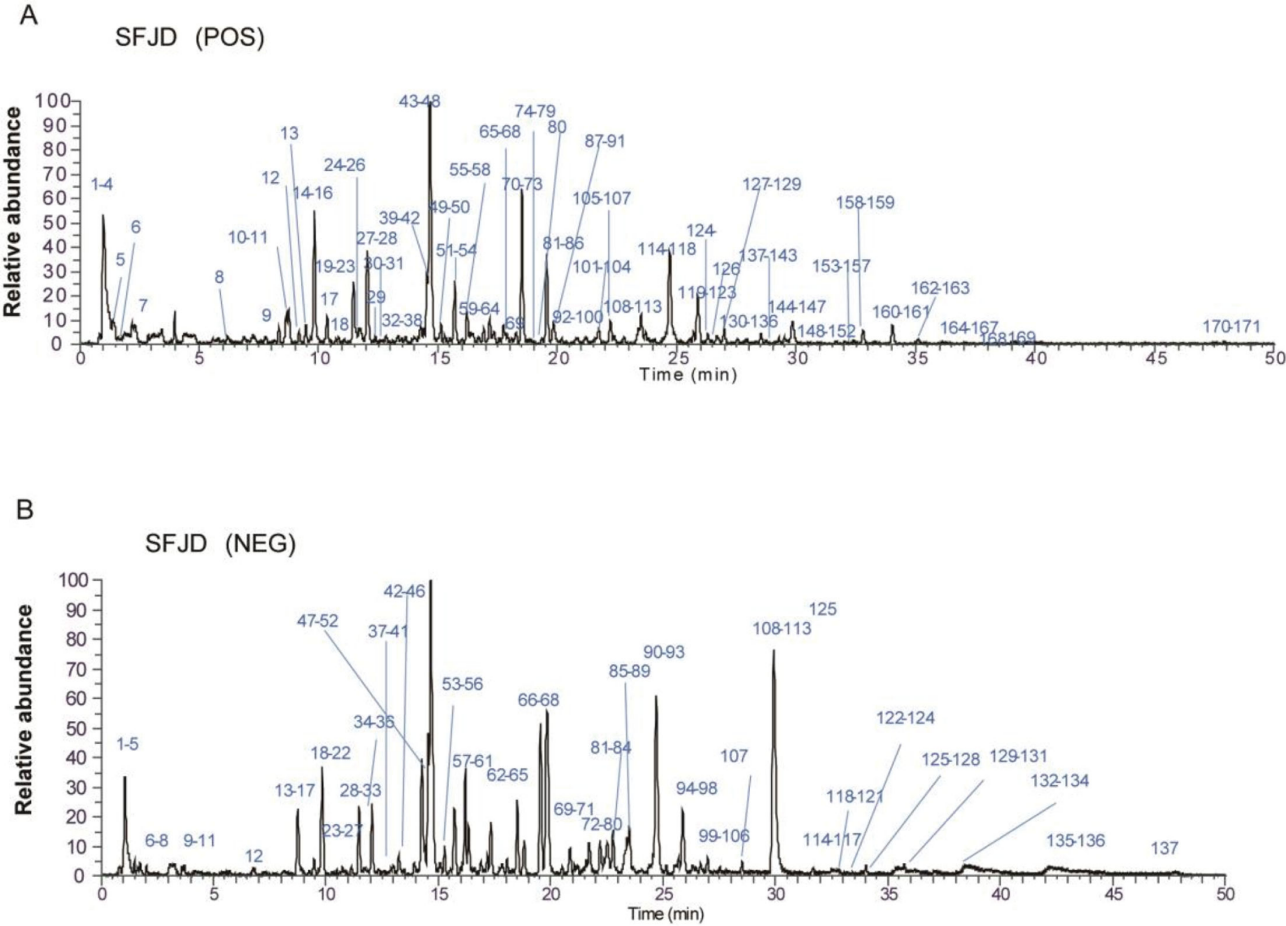
Chemical composition analysis. (**A**) Positive ion mode, (**B**) negative ion mode.

SFJD is a commercially available patent medicine, which has demonstrated significant efficacy in the intervention and treatment of diseases during the COVID-19 pandemic (24, 25) To elucidate the principal chemical constituents of SFJD, we employed ultra-performance liquid chromatography coupled with quadrupole exactive orbitrap mass spectrometry (UPLC-Q-Exactive-Orbitrap MS) for a thorough analysis. The data obtained were processed utilizing Thermo Xcalibur 4.1 and Compound Discoverer 3.2 software, resulting in the identification of a total of 171 chemical constituents through mass spectrometry fragment ion analysis, database matching, and comparisons with pertinent literature (Fig. 4A and B). The predominant components identified in SFJD encompassed flavonoids, glycosides, terpenoids, phenylpropanoids, alkaloids, organic acids, anthraquinones, among others (Table S1).

### SFJD reduces host cell susceptibility to H1N1, RSV, and 229E viruses

**Fig 5:**
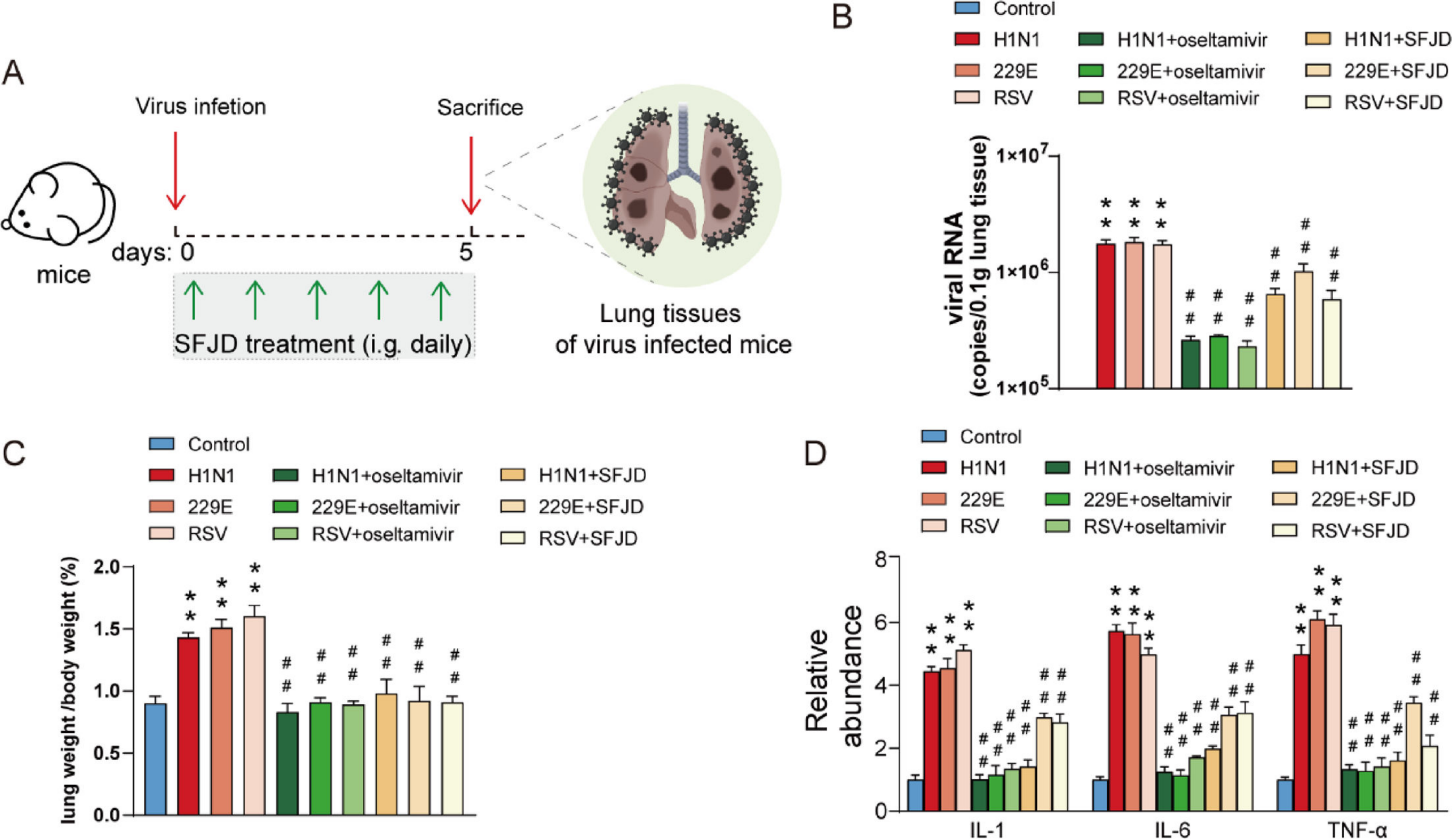
The effect of SFJD capsules on the H1N1, RSV, and 229E virus. (**A**) Animal modeling and drug delivery. (**B**) Viral RNA levels in the lung tissue of mice. Lung index (**C**) and inflammatory markers level in the lung tissue (**D**) of mice. Mice were infected with H1N1, RSV, and 229E viruses and treated with positive drug oseltamivir or SFJD via gavage. Animal viral infections dosage = 100 TCID50. *t*-test, **P < 0.01, model group (virus-infected) vs control group; *#P* < 0.05, *##P* < 0.01, medication group vs model group, *n* = 6.

In order to assess the effect of SFJD on the H1N1, RSV, and 229E virus after invasion of mouse lungs (Fig. 5A), we examined the expression of viral RNA levels, lung index, and inflammatory factors, such as IL-1, IL-6, and TNF-α in mouse lung tissues. As shown in Fig. 5B, the model group showed a significant raise in viral RNA levels of lung tissue when compared with the control group. Compared with the model group, both the positive drug oseltamivir and the SFJD group showed a significant decrease in viral RNA levels. This suggests that SFJD can reduce the invasion efficiency of the virus into the host. In comparison to the model group, the group administered the SFJD exhibited reduction in lung index (Fig. 5C). As shown in Fig. 5D, the model group exhibits higher levels of IL-1, IL-6, and TNF-α compared with the control group. Whereas, the levels of IL-1, IL-6, and TNF-α were significantly reduced upon the administration of the positive drug oseltamivir and the SFJD. The above results suggest that SFJD can attenuate the susceptibility of host cells to the virus and improve the outcome of infection satisfactorily.

### SFJD reduces viral susceptibility of host cells by interfering with SLC16A3-AP1G1 interactions

**Fig 6:**
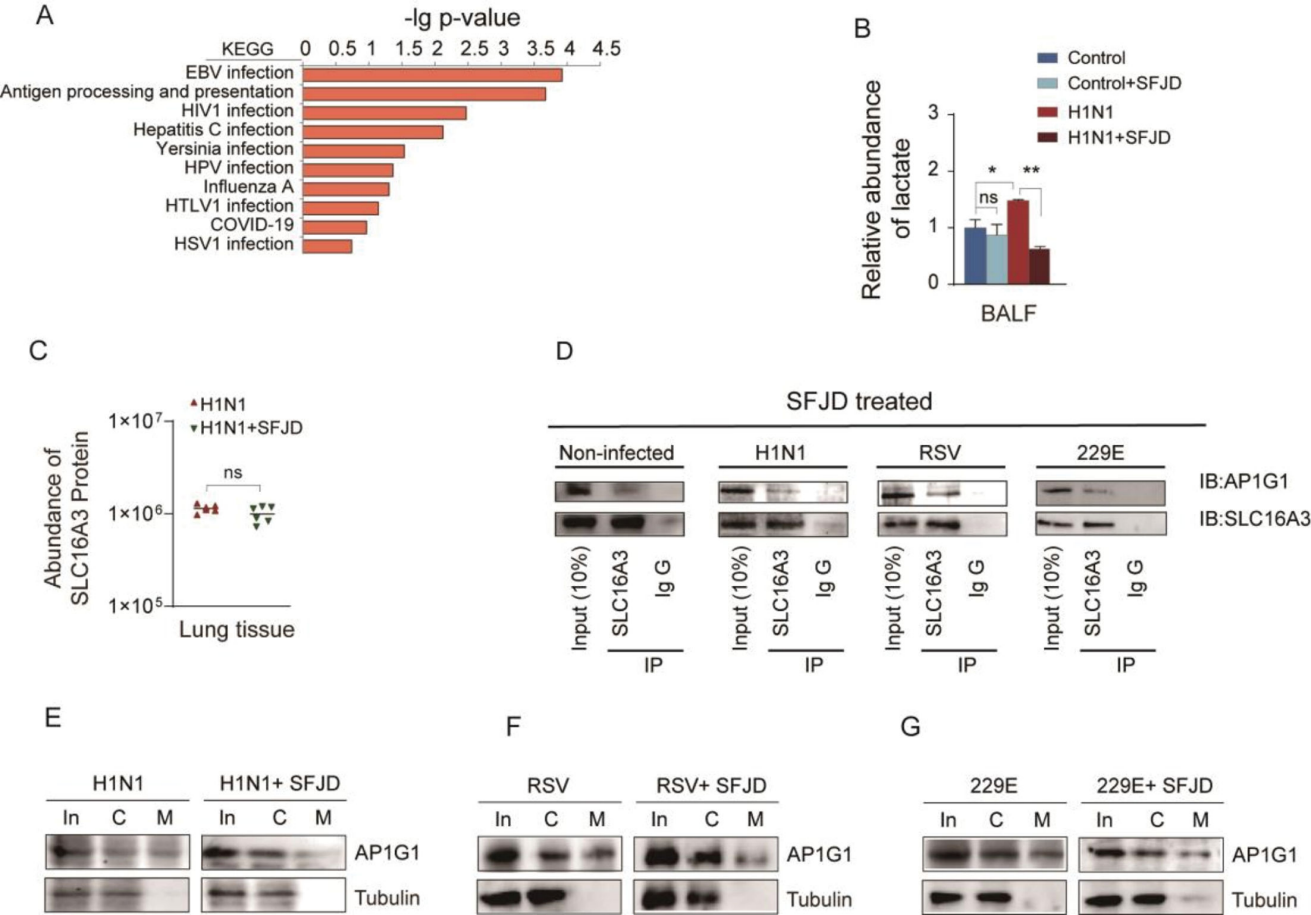
SFJD intervention with SLC16A3-AP1G1 interaction. (**A**) KEGG Strip Pathway. (**B**) Lactate expression levels in BALF of mice before and after SFJD administration after infection with H1N1 virus. *t*-test, *P < 0.05, **P < 0.01, *n* = 6. (**C**) SLC16A3 expression levels in lung tissue of mice before and after SFJD administration after infection with H1N1 virus (*n* = 6). (**D**) IP-WB assay illustrating the attenuated interaction between SLC16A3 and AP1G1 following SFJD treatment (*n* = 3). (**E–G**) Subcellular localization of AP1G1 before and after SFJD administration following infection with H1N1, RSV, and 229E viruses. Cells were infected with H1N1, RSV, and 229E virus (MOI = 2, *n* = 3).

Proteomic analyses indicated that the administration of SFJD influenced the biological processes associated with various viral infections (Fig. 6A). These findings imply that SFJD exerts its anti-virus effect through a general mechanism. Apart from reducing viral load of lung tissue (Fig. 5B), SFJD was also effective in reducing lactate abundance in the bronchoalveolar lavage fluid (BALF) (Fig. 6B). However, SFJD did not lead to a reduction in the expression levels of SLC16A3 (Fig. 6C). Interestingly, IP-WB results demonstrated that SFJD administration diminished the interaction between SLC16A3 and AP1G1 (Fig. 6D). SFJD also inhibited the accumulation of AP1G1 at the cellular membrane (Fig. 6E through G). These results indicated that SFJD attenuates the membrane enrichment of AP1G1, thereby impairing its functional capacity in endocytosis, which mediates virus invasion. Collectively, these results suggest that SFJD impairs the SLC16A3-AP1G1 interaction, thus inhibiting the localization of AP1G1 at the cell membrane and ultimately reducing the susceptibility of host cells (evaluated by endocytosis capacity) to a diverse array of viral pathogens.

## DISCUSSION

Host metabolic processes play a crucial role in the progression of viral infections. The metabolic status of the host not only impacts viral replication and transmission but also has a close connection to the host’s immune response (26, 27). The clathrin adaptor protein complex-1 (AP-1) is one of the key players in cellular transport mechanisms that can influence viral infections by determining the fate of various cellular cargos, including receptors and other proteins involved in viral entry and replication (28). Research indicates that respiratory virus infections can significantly modify host metabolic pathways. For instance, respiratory viruses like influenza and SARS-CoV-2 can disrupt lactate metabolic pathways, leading to increased lactate production (29). The inflammatory and immune responses triggered by viral infections can also result in heightened lactate levels (30). Furthermore, lactate levels can serve as indicators for assessing the prognosis of patients with respiratory viral infections, with high lactate levels often correlating with increased mortality rates (31). Nevertheless, there is still a lack of comprehensive understanding regarding how host metabolic processes, including lactate and related metabolic proteins, reciprocally influence viral invasion mechanisms.

In this study, it was observed that lactate levels were significantly increased in the lung serum of H1N1 virus-infected mice, consistent with previous research (32, 33). Similarly, elevated levels of lactate were detected in the cell body and medium of BEAS2B cells infected with the virus. The impact of reducing lactate levels through 2-DG treatment was examined to assess whether modulation of lactate expression could hinder viral invasion. However, it was found that the decreased lactate level did not lead to suppression on viral invasion, which indicates that the concentration of lactate is not a decisive factor for host cell susceptibility. Analysis of cell samples post-viral infection using proteomics revealed a significant alteration in the expression of the lactate transporter SLC16A3. SLC16A3 plays an important role in the transport of lactate and is critical for maintaining cellular energy metabolism, which could affect viral replication processes and host immune responses to infections (28). SLC16A3 is responsible for the transport of lactate, and its upregulation can be achieved by regulating cellular metabolism and acidic environment (26), which may affect the process of viral replication and infection. In addition, SLC16A3 regulation may be associated with host immune responses (34), which may also influence the outcome of viral infection. Here we found that higher expression of SLC16A3 was observed in cells with heightened viral susceptibility, and knockdown of SLC16A3 resulted in reduced viral load in host cells. Therefore, SLC16A3 plays a crucial role in the viral infection process.

AP1G1 plays a crucial role in viral infection. As detailed in previous studies, AP1G1 is critical for the endocytosis of viruses, promoting their entry into host cells by facilitating the clathrin-mediated trafficking pathways essential for viral cellular uptake (28). As a component of the AP-1 complex, AP1G1 is involved in the endosome-Golgi transport process, influencing viral invasion and intracellular transport (35, 36). Simultaneously, it regulates the endocytosis and transportation of viral particles, impacting the efficiency of viral replication. Thus, AP1G1 plays an important role in viral invasion, transport, replication, and immune evasion. It is a key host factor in the infection process of viruses such as SARS-CoV-2 (37). Our study further explored the mechanism linking SLC16A3 to viral susceptibility. We found that SLC16A3 and AP1G1 interact and are highly co-localized in cells. Enrichment in the cell membrane is essential for AP1G1 to participate in endocytosis activity in cells (38). We found that the knockdown of SLC16A3 led to a significant decrease in AP1G1 enrichment at the cell membrane, resulting in a subsequent reduction of viral load in the cells. To sum up, SLC16A3 plays a crucial role in the migratory enrichment of AP1G1 to the cell membrane, influencing host cell viral susceptibility by changing the cellular sublocalization of AP1G1.

Moreover, our findings are consistent with the notion that metabolic pathways altered during viral infections can impact host factor localization. This is particularly relevant considering that AP-1’s diverse roles in vesicle trafficking are fundamental for the proper localization of viral receptors and related proteins, thereby directly affecting viral entry into host cells (28). Furthermore, the interplay between SLC16A3 and AP1G1 emphasizes the importance of metabolic regulation in the context of viral infections, highlighting how host cellular environment can dictate viral uptake efficiency (38). The evidence from previous studies highlights the complex interplay between metabolism and viral entry mechanisms, with specific host factors like SLC16A3 contributing to changes in cellular microenvironments that facilitate viral invasion (37).

It is important to note that TCM demonstrates inhibitory effects against a variety of viruses and variola infections, suggesting the possibility of a broad-spectrum antiviral mechanism (39–42). TCM ingredients, such as D flavonoids (43, 44), myricetin (45, 46), and quercetin (47, 48), exhibit significant antiviral activity (49–51). These previous studies strongly supported us in prompting us to take a deep insight into the effect of SFJD on host cell viral susceptibility and its potential antiviral mechanisms in this study. Our results suggest that the modulation of host metabolic pathways and the regulation of key proteins like SLC16A3 and AP1G1 could be potential therapeutic strategies for controlling viral infections (37). As our expectation, SFJD significantly reduced the levels of viral RNA, lung index, and inflammatory factors, such as IL-1, IL-6, and TNF-α, in lung tissues of H1N1 virus-infected mice. At the cellular level, SFJD was found to have a significant broad-spectrum inhibition of viral invasion and a reduction of host cell susceptibility to a variety of viruses. Regarding regulatory mechanisms, SFJD treatment could not reduce the expression of SLC16A3 but effectively reduced lactate exocytosis in animal alveoli. IP-WB analysis showed that the interaction between SLC16A3 and AP1G1 was attenuated after SFJD treatment. Additionally, the membrane enrichment of AP1G1 in SFJD-treatment cells was significantly reduced. Thus, SFJD affects the membrane localization of AP1G1, thereby suppressing the cell endocytosis of viral particles and decreasing the host susceptibility.

### Conclusion

This study identified the disruption of the SLC16A3-AP1G1 interaction as a pathway that modulates host susceptibility for antiviral purposes with a broad spectrum. The feasibility of this regulatory mechanism was also demonstrated in SFJD. This study offers new insights into the mechanism of regulating host cell metabolic activities to inhibit viral infection and provides valuable reference points for the exploration of antiviral drug targets.

## Data Availability

The metabolomics data generated and analyzed in this study have been deposited in the MetaboLights database with the accession number MTBLS12788; the proteomics data have been submitted to the ProteomeXchange Consortium (PXC) via the iProX partner repository, with the dataset identifier PXD066638. Both types of datasets are publicly accessible, without any access restrictions or expiration dates. Researchers can obtain the data for further research by submitting an application through the specified channels

## SUPPLEMENTAL MATERIAL

Supplemental Material spectrum.03116-24-s0001.docx Figures S1 and S2; Table S1.

ASM does not own the copyrights to Supplemental Material that may be linked to, or accessed through, an article. The authors have granted ASM a non-exclusive, world-wide license to publish the Supplemental Material files. Please contact the corresponding author directly for reuse.
