# Supplementary material for: Attenuate host susceptibility to respiratory virus invasion by inhibiting interactions between host proteins SLC16A3 and AP1G1
Source: Microbiol Spectr. 2025 Sep 8;13(10):e03116-24. doi: 10.1128/spectrum.03116-24 (PMC12502636; doi:10.1128/spectrum.03116-24)
Supplement: Supplemental Material — Figures S1 and S2; Table S1. [file spectrum.03116-24-s0001.docx]

Supplementary Material

#
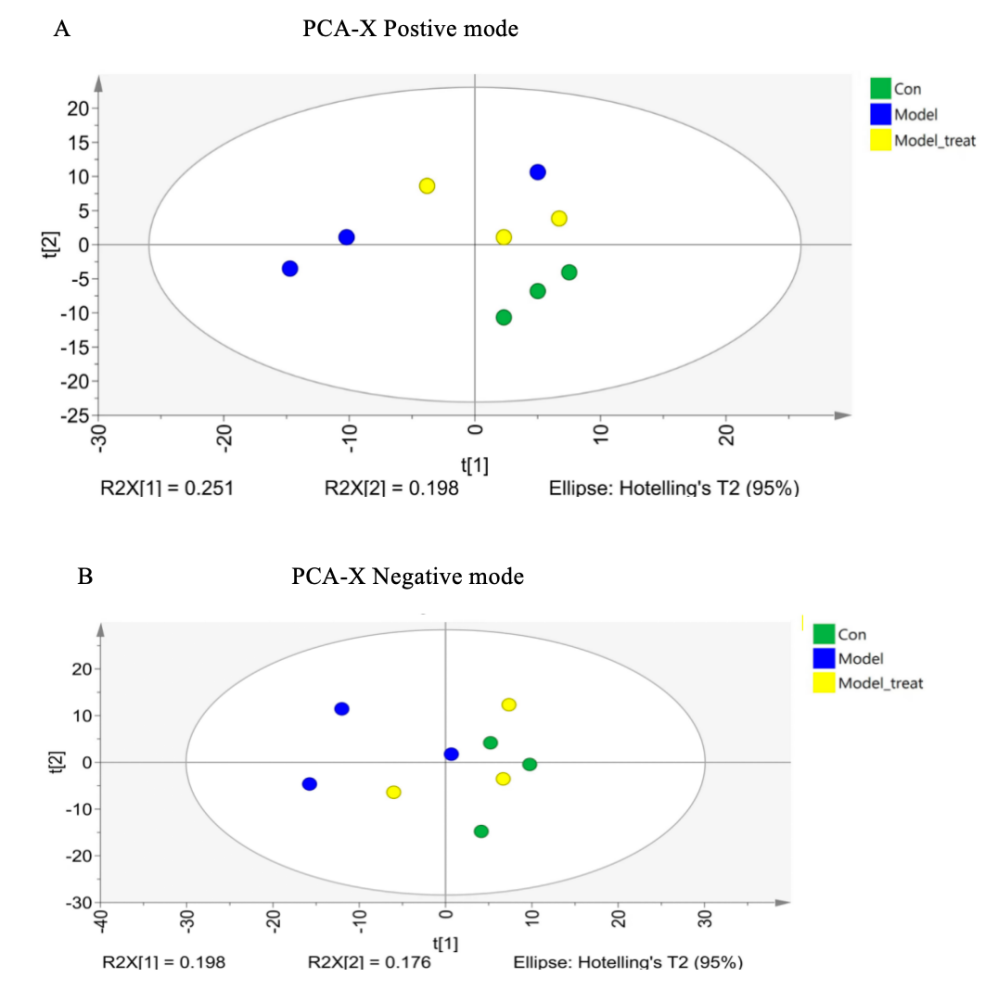


**Supplementary Figure 1. PCA analysis based on data obtained from positive (A) and negative (B) on mode.**

**Supplementary Figure 2. rt-qPCR results illustrating the knock-down efficacy of the SLC16A3 with the shRNA**

**Table S1 UPLC-QTOF-MS/MS Identification Results of Compounds in SFJD**

| No | identity | Molecular formula | Error（ppm） | Observed mass  (*m/z*) | Calculated mass  (*m/z*) | RT （min） | mzCloud Results | mzVault Results | mzCloud Best Match | mzCloud Best  Match Confidence | mzVault Best Match | MS Depth | Type of chemical composition |
| --- | --- | --- | --- | --- | --- | --- | --- | --- | --- | --- | --- | --- | --- |
| 1 | Maltotetraose | C_24_H_42_O_21_ | 32991.42 | 666.22186 | 688.20146 | 1.032 | 1 | 0 | 90.9 | 89.4 |  | 2 | Saccharides |
| 2 | D-Raffinose | C_18_H_32_O_16_ | -4.46 | 504.16903 | 504.16679 | 1.06 | 1 | 5 | 87.3 | 9.4 | 69 | 2 | Saccharides |
| 3 | D-(+)-Maltose | C_12_H_22_O_11_ | 64250.11 | 342.11621 | 364.09722 | 1.123 | 3 | 0 | 84.7 | 9.2 |  | 2 | Saccharides |
| 4 | α-Lactose | C_12_H_22_O_11_ | 49763.73 | 342.11621 | 359.14119 | 1.208 | 2 | 16 | 84.4 | 33.1 | 88.4 | 2 | Saccharides |
| 5 | L-Prolyl-L-isoleucine | C_11_H_20_N_2_O_3_ | -3.7 | 228.14739 | 228.14655 | 1.616 | 2 | 0 | 81.7 | 9.1 |  | 2 | Amino acid |
| 6 | Adenosine | C_10_H_13_N_5_O_4_ | -3.98 | 267.09675 | 267.09569 | 2.301 | 3 | 0 | 90.1 | 9.5 |  | 2 | Alkaloids |

**Continued Table S1 UPLC-QTOF-MS/MS Identification Results of Compounds in SFJD**

| No | identity | Molecular formula | Error（ppm） | Observed mass  (m/z) | Calculated mass  (m/z) | RT （min） | mzCloud Results | mzVault Results | mzCloud Best Match | mzCloud Best  Match Confidence | mzVault Best Match | MS Depth | Type of chemical composition |
| --- | --- | --- | --- | --- | --- | --- | --- | --- | --- | --- | --- | --- | --- |
| 7 | 2'-Deoxyadenosine | C_10_H_13_N_5_O_3_ | -3.71 | 251.10184 | 251.10091 | 2.719 | 6 | 3 | 82.5 | 9.1 | 90.5 | 2 |  |
| 8 | Cornoside | C_14_H_20_O_8_ | -4.04 | 316.11582 | 316.11454 | 6.88 | 0 | 0 |  |  |  | 2 | Phenols |
| 9 | DL-Tryptophan | C_11_H_12_N_2_O_2_ | -3.82 | 204.08988 | 204.0891 | 8.741 | 3 | 0 | 89.1 | 36 |  | 2 | Alkaloids |
| 10 | 4-(2-Hydroxyethyl)-2-methoxyphenyl β-D-glucopyranoside | C_15_H_22_O_8_ | -4.01 | 330.13147 | 330.13014 | 9.077 | 1 | 0 | 80.4 | 9 |  | 2 | glycoside |
| 11 | 5'-S-Methyl-5'-thioadenosine | C_11_H_15_N_5_O_3_S | -3.51 | 297.08956 | 297.08852 | 9.098 | 1 | 0 | 91.5 | 9.6 |  | 2 | glycoside |

**Continued Table S1 UPLC-QTOF-MS/MS Identification Results of Compounds in SFJD**

| **No** | **identity** | **Molecular formula** | **Error（ppm）** | **Observed mass**  **(m/z)** | **Calculated mass**  **(m/z)** | **RT （min）** | **mzCloud Results** | **mzVault Results** | **mzCloud Best Match** | **mzCloud Best**  **Match Confidence** | **mzVault Best Match** | **MS Depth** | **Type of chemical composition** |
| --- | --- | --- | --- | --- | --- | --- | --- | --- | --- | --- | --- | --- | --- |
| 12 | (1R,9S)-5-(1-Benzothiophen-2-yl)-11-(cyclohexylcarbonyl)-7,11-diazatricyclo[7.3.1.02,7]trideca-2,4-dien-6-one | C_26_H_28_N_2_O_2_S | 87811.33 | 432.18715 | 470.13808 | 9.458 | 1 | 0 | 84 | 9.2 |  | 2 |  |
| 13 | Gibboside | C_16_H_26_O_9_ | -4.5 | 362.15768 | 362.15605 | 9.672 | 0 | 0 |  |  |  | 2 | Triterpenoids |
| 14 | Isaindigodione | C_18_H_18_N_2_O_4_ | -3.48 | 326.12666 | 326.12552 | 10.093 | 0 | 0 |  |  |  | 2 |  |
| 15 | Coniferin | C_16_H_22_O_8_ | 49762.24 | 342.13147 | 359.15669 | 10.127 | 1 | 0 | 87.5 | 35 |  | 2 | Phenylpropanoids |
| 16 | 1,6-Bis-O-(3,4,5-trihydroxybenzoyl)hexopyranose | C_20_H_20_O_14_ | -37208.54 | 484.08531 | 466.0732 | 10.539 | 1 | 0 | 86.3 | 9.3 |  | 2 |  |

**Continued Table S1 UPLC-QTOF-MS/MS Identification Results of Compounds in SFJD**

| No | identity | Molecular formula | Error（ppm） | Observed mass  (m/z) | Calculated mass  (m/z) | RT （min） | mzCloud Results | mzVault Results | mzCloud Best Match | mzCloud Best  Match Confidence | mzVault Best Match | MS Depth | Type of chemical composition |
| --- | --- | --- | --- | --- | --- | --- | --- | --- | --- | --- | --- | --- | --- |
| 17 | 2,3,4,9-Tetrahydro-1H-β-carboline-3-carboxylic acid | C_12_H_12_N_2_O_2_ | -2.76 | 216.08988 | 216.08928 | 10.555 | 1 | 0 | 89.4 | 9.5 |  | 2 | Alkaloids |
| 18 | Catechin | C_15_H_14_O_6_ | -4.25 | 290.07904 | 290.07781 | 10.823 | 3 | 0 | 91.9 | 41.9 |  | 2 | Flavonoids |
| 19 | 6-O-(2-Methylbutanoyl)-α-D-glucopyranosyl α-D-glucopyranoside | C_17_H_30_O_12_ | 51577.02 | 426.17373 | 448.1545 | 11.219 | 1 | 0 | 81.2 | 31.2 |  | 2 | glycoside |

**Continued Table S1 UPLC-QTOF-MS/MS Identification Results of Compounds in SFJD**

| No | identity | Molecular formula | Error（ppm） | Observed mass  (m/z) | Calculated mass  (m/z) | RT （min） | mzCloud Results | mzVault Results | mzCloud Best Match | mzCloud Best  Match Confidence | mzVault Best Match | MS Depth | Type of chemical composition |
| --- | --- | --- | --- | --- | --- | --- | --- | --- | --- | --- | --- | --- | --- |
| 20 | Methyl (1S,7R)-1-(β-D-glucopyranosyloxy)-7-methyl-5-oxo-1,4a,5,6,7,7a-hexahydrocyclopenta[c]pyran-4-carboxylate | C_17_H_24_O_10_ | -3.69 | 388.13695 | 388.13552 | 11.34 | 2 | 0 | 88.6 | 35.7 |  | 2 | Triterpenoids |
| 21 | Tachiogroside B | C_18_H_24_O_12_ | -3.8 | 432.12678 | 432.12513 | 11.347 | 0 | 0 |  |  |  | 2 | Flavonoids |
| 22 | Quercetin-3β-D-glucoside | C_21_H_20_O_12_ | -3.65 | 464.09548 | 464.09378 | 11.372 | 2 | 0 | 86.1 | 9.3 |  | 2 | Flavonoids |

**Continued Table S1 UPLC-QTOF-MS/MS Identification Results of Compounds in SFJD**

| **No** | **identity** | **Molecular formula** | **Error（ppm）** | **Observed mass**  **(m/z)** | **Calculated mass**  **(m/z)** | **RT （min）** | **mzCloud Results** | **mzVault Results** | **mzCloud Best Match** | **mzCloud Best**  **Match Confidence** | **mzVault Best Match** | **MS Depth** | **Type of chemical composition** |
| --- | --- | --- | --- | --- | --- | --- | --- | --- | --- | --- | --- | --- | --- |
| 23 | Methyl 1-(hexopyranosyloxy)-4a-hydroxy-7-methyl-5-oxo-1,4a,5,6,7,7a-hexahydrocyclopenta[c]pyran-4-carboxylate | C_17_H_24_O_11_ | -4.29 | 404.13186 | 404.13013 | 11.455 | 1 | 0 | 94.5 | 83.5 |  | 2 |  |
| 24 | Aesculetin dimethyl ether | C_11_H_10_O_4_ | -3.33 | 206.05791 | 206.05722 | 11.455 | 4 | 0 | 75.6 | 47.1 |  | 2 | Coumarins |
| 25 | Sinapyl alcohol | C_11_H_14_O_4_ | -3.43 | 210.08921 | 210.08849 | 11.694 | 0 | 0 |  |  |  | 2 | Phenylpropanoids |
| 26 | Loganin | C_17_H_26_O_10_ | 56337.76 | 390.1526 | 412.13292 | 11.695 | 1 | 0 | 84.3 | 33.1 |  | 2 | Triterpenoids |

**Continued Table S1 UPLC-QTOF-MS/MS Identification Results of Compounds in SFJD**

| **No** | **identity** | **Molecular formula** | **Error（ppm）** | **Observed mass**  **(m/z)** | **Calculated mass**  **(m/z)** | **RT （min）** | **mzCloud Results** | **mzVault Results** | **mzCloud Best Match** | **mzCloud Best**  **Match Confidence** | **mzVault Best Match** | **MS Depth** | **Type of chemical composition** |
| --- | --- | --- | --- | --- | --- | --- | --- | --- | --- | --- | --- | --- | --- |
| 27 | Cornin | C_17_H_24_O_10_ | -4.45 | 388.13695 | 388.13522 | 12.031 | 4 | 0 | 91.3 | 40.2 |  | 2 | Glycoside |
| 28 | Brasoside | C_16_H_22_O_9_ | -3.95 | 358.12638 | 358.12497 | 12.139 | 0 | 0 |  |  |  | 2 |  |
| 29 | Berberine | C_20_H_17_NO_4_ | -3.61 | 335.11576 | 335.11455 | 12.407 | 1 | 0 | 83.3 | 9.2 |  | 2 | Alkaloids |
| 30 | Schaftoside | C_26_H_28_O_14_ | -3.7 | 564.14791 | 564.14582 | 12.675 | 2 | 0 | 84 | 78.7 |  | 2 | Flavonoids |
| 31 | Piceatannol | C_14_H_12_O_4_ | -3.23 | 244.07356 | 244.07277 | 12.913 | 1 | 0 | 84.3 | 79.2 |  | 2 | Phenols |
| 32 | Quercetin 3-O-rhamnoside-7-O-glucoside | C_27_H_30_O_16_ | -3.03 | 610.15338 | 610.15154 | 13.12 | 2 | 0 | 82.2 | 31.8 |  | 2 | Flavonoids |
| 33 | Corymboside | C_26_H_28_O_14_ | -3.21 | 564.14791 | 564.14609 | 13.386 | 2 | 0 | 89.2 | 86.3 |  | 2 | Flavonoids |
| 34 | Isatan B | C_14_H_15_NO_6_ | -3.49 | 293.08994 | 293.08891 | 13.437 | 0 | 0 |  |  |  | 2 |  |

**Continued Table S1 UPLC-QTOF-MS/MS Identification Results of Compounds in SFJD**

| No | identity | Molecular formula | Error（ppm） | Observed mass  (m/z) | Calculated mass  (m/z) | RT （min） | mzCloud Results | mzVault Results | mzCloud Best Match | mzCloud Best  Match Confidence | mzVault Best Match | MS Depth | Type of chemical composition |
| --- | --- | --- | --- | --- | --- | --- | --- | --- | --- | --- | --- | --- | --- |
| 35 | Robinin | C_33_H_40_O_19_ | -3.51 | 740.21638 | 740.21378 | 13.734 | 2 | 0 | 88.2 | 9.4 |  | 2 | Flavonoids |
| 36 | 2-Oxo-2H-chromen-7-yl 6-O-β-D-xylopyranosyl-β-D-glucopyranoside | C_20_H_24_O_12_ | -3.86 | 456.12678 | 456.12502 | 14.401 | 1 | 0 | 81.3 | 9.1 |  | 2 |  |
| 37 | 2-(3,4-Dihydroxyphenyl)ethyl pentopyranosyl-(1->2)-6-deoxyhexopyranosyl-(1->3)-4-O-[(2E)-3-(3,4-dihydroxyphenyl)-2-propenoyl]hexopyranoside | C_34_H_44_O_19_ | 22510.22 | 756.24768 | 773.27098 | 14.424 | 1 | 0 | 86.3 | 9.3 |  | 2 | Triterpenoids |

**Continued Table S1 UPLC-QTOF-MS/MS Identification Results of Compounds in SFJD**

| No | identity | Molecular formula | Error（ppm） | Observed mass  (m/z) | Calculated mass  (m/z) | RT （min） | mzCloud Results | mzVault Results | mzCloud Best Match | mzCloud Best  Match Confidence | mzVault Best Match | MS Depth | Type of chemical composition |
| --- | --- | --- | --- | --- | --- | --- | --- | --- | --- | --- | --- | --- | --- |
| 38 | Glycitin | C_22_H_22_O_10_ | -4.11 | 446.1213 | 446.11946 | 14.458 | 2 | 0 | 83.6 | 9.2 |  | 2 | Flavonoids |
| 39 | (E)-Piceid | C_20_H_22_O_8_ | -4.13 | 390.13147 | 390.12986 | 14.538 | 0 | 0 |  |  |  | 2 | Anthraquinones |
| 40 | Resveratrol | C_14_H_12_O_3_ | -3.41 | 228.07864 | 228.07787 | 14.547 | 6 | 0 | 95.1 | 98.2 |  | 2 | Phenols |
| 41 | Liquiritigenin | C_15_H_12_O_4_ | -3.79 | 256.07356 | 256.07259 | 14.574 | 2 | 0 | 92.6 | 93.1 |  | 2 | Flavonoids |
| 42 | Quercetin | C_15_H_10_O_7_ | -4.25 | 302.04265 | 302.04137 | 14.583 | 3 | 0 | 89.4 | 36.2 |  | 2 | Flavonoids |
| 43 | Rutin | C_27_H_30_O_16_ | -3.85 | 610.15338 | 610.15104 | 14.591 | 2 | 0 | 85.7 | 9.3 |  | 2 | Flavonoids |
| 44 | Lariciresinol 4-O-glucoside | C_26_H_34_O_11_ | 32600.97 | 522.21011 | 539.23467 | 14.592 | 1 | 0 | 88.3 | 35.5 |  | 2 | Lignans |

**Continued Table S1 UPLC-QTOF-MS/MS Identification Results of Compounds in SFJD**

| **No** | **identity** | **Molecular formula** | **Error（ppm）** | **Observed mass**  **(m/z)** | **Calculated mass**  **(m/z)** | **RT （min）** | **mzCloud Results** | **mzVault Results** | **mzCloud Best Match** | **mzCloud Best**  **Match Confidence** | **mzVault Best Match** | **MS Depth** | **Type of chemical composition** |
| --- | --- | --- | --- | --- | --- | --- | --- | --- | --- | --- | --- | --- | --- |
| 45 | 2-(3,4-Dihydroxyphenyl)ethyl 3-O-(6-deoxy-β-L-mannopyranosyl)-6-O-[(2E)-3-(3,4-dihydroxyphenyl)-2-propenoyl]-β-D-glucopyranoside | C_29_H_36_O_15_ | 27273.26 | 624.20542 | 641.22954 | 14.66 | 6 | 0 | 87.2 | 9.4 |  | 2 |  |
| 46 | Catechin gallate | C_22_H_18_O_10_ | -7.76 | 442.09 | 442.08656 | 14.818 | 1 | 0 | 88.3 | 9.4 |  | 2 | Flavonoids |
| 47 | Butein | C_15_H_12_O_5_ | -3.61 | 272.06847 | 272.06749 | 14.829 | 2 | 0 | 71.9 | 25.5 |  | 2 | Flavonoids |
| 48 | Homosildenafil | C_23_H_32_N_6_O_4_S | 3.24 | 488.22057 | 488.22215 | 15.304 | 5 | 0 | 80.6 | 9 |  | 2 |  |

**Continued Table S1 UPLC-QTOF-MS/MS Identification Results of Compounds in SFJD**

| No | identity | Molecular formula | Error（ppm） | Observed mass  (m/z) | Calculated mass  (m/z) | RT （min） | mzCloud Results | mzVault Results | mzCloud Best Match | mzCloud Best  Match Confidence | mzVault Best Match | MS Depth | Type of chemical composition |
| --- | --- | --- | --- | --- | --- | --- | --- | --- | --- | --- | --- | --- | --- |
| 49 | Citrinin | C_13_H_14_O_5_ | -72021.45 | 250.08412 | 232.0727 | 15.37 | 1 | 0 | 85.9 | 9.3 |  | 2 | Flavonoids |
| 50 | 2-Phenylethyl 3-O-(4-carboxy-3-hydroxy-3-methylbutanoyl)-β-D-glucopyranoside | C_20_H_28_O_10_ | 39762.42 | 428.16825 | 445.19325 | 15.458 | 1 | 0 | 81.6 | 9.1 |  | 2 |  |
| 51 | Kaempferol-3-O-β-glucopyranosyl-7-O-α-rhamnopyranoside | C_27_H_30_O_15_ | -3.45 | 594.15847 | 594.15642 | 15.642 | 3 | 0 | 83.8 | 9.2 |  | 2 | Flavonoids |

**Continued Table S1 UPLC-QTOF-MS/MS Identification Results of Compounds in SFJD**

| No | identity | | Molecular formula | Error（ppm） | Observed mass  (m/z) | Calculated mass  (m/z) | RT （min） | mzCloud Results | mzVault Results | mzCloud Best Match | mzCloud Best  Match Confidence | mzVault Best Match | MS Depth | Type of chemical composition |
| --- | --- | --- | --- | --- | --- | --- | --- | --- | --- | --- | --- | --- | --- | --- |
| 52 | 4-[4-(4-Hydroxy-3-methoxyphenyl)tetrahydro-1H,3H-furo[3,4-c]furan-1-yl]-2-methoxyphenyl hexopyranoside | | C_26_H_32_O_11_ | 32727.22 | 520.19446 | 537.21898 | 15.688 | 2 | 0 | 88.3 | 64 |  | 2 | Lignans |
| 53 | Pinoresinol | C_20_H_22_O_6_ | | -50292.82 | 358.14164 | 340.12969 | 15.697 | 1 | 0 | 69.8 | 58.3 |  | 2 | Lignans |
| 54 | Glycyuralin E | C_21_H_22_O_6_ | | -3.93 | 370.14164 | 370.14018 | 16.04 | 0 | 0 |  |  |  | 2 | Flavonoids |

**Continued Table S1 UPLC-QTOF-MS/MS Identification Results of Compounds in SFJD**

| No | identity | Molecular formula | Error（ppm） | Observed mass  (m/z) | Calculated mass  (m/z) | RT （min） | mzCloud Results | mzVault Results | mzCloud Best Match | mzCloud Best  Match Confidence | mzVault Best Match | MS Depth | Type of chemical composition |
| --- | --- | --- | --- | --- | --- | --- | --- | --- | --- | --- | --- | --- | --- |
| 55 | 8-Prenylnaringenin | C_20_H_20_O_5_ | -4.08 | 340.13107 | 340.12969 | 16.219 | 0 | 0 |  |  |  | 2 | Flavonoids |
| 56 | Resveratrol | C_14_H_12_O_3_ | -3 | 228.07864 | 228.07796 | 16.342 | 2 | 0 | 95.6 | 98.3 |  | 2 | Phenols |
| 57 | Neodiosmin | C_28_H_32_O_15_ | -3.61 | 608.17412 | 608.17193 | 16.369 | 2 | 0 | 82.6 | 9.1 |  | 2 | Flavonoids |
| 58 | Apigenin 7-O-glucuronide | C_21_H_18_O_11_ | -3.54 | 446.08491 | 446.08333 | 16.476 | 3 | 0 | 81.8 | 9.1 |  | 2 | Flavonoids |
| 59 | Questin | C_16_H_12_O_5_ | -3.99 | 284.06847 | 284.06734 | 16.51 | 7 | 0 | 88.1 | 9.4 |  | 2 | Anthraquinones |

**Continued Table S1 UPLC-QTOF-MS/MS Identification Results of Compounds in SFJD**

| No | identity | Molecular formula | Error（ppm） | Observed mass  (m/z) | Calculated mass  (m/z) | RT （min） | mzCloud Results | mzVault Results | mzCloud Best Match | mzCloud Best  Match Confidence | mzVault Best Match | MS Depth | Type of chemical composition |
| --- | --- | --- | --- | --- | --- | --- | --- | --- | --- | --- | --- | --- | --- |
| 60 | 4,5-Dicaffeoylquinic acid | C_25_H_24_O_12_ | -3.31 | 516.12678 | 516.12507 | 16.615 | 1 | 0 | 79.4 | 9 |  | 2 | Phenylpropanoids |
| 61 | Kuromanin | C_21_H_20_O_11_ | -3.65 | 448.10056 | 448.09892 | 16.688 | 3 | 0 | 81 | 9.1 |  | 2 | Flavonoids |
| 62 | Fisetin | C_15_H_10_O_6_ | -3.38 | 286.04774 | 286.04677 | 16.822 | 3 | 0 | 72.6 | 8.6 |  | 2 | Flavonoids |
| 63 | 6-O-Methylscutellarin | C_22_H_20_O_12_ | -3.57 | 476.09548 | 476.09378 | 16.909 | 1 | 0 | 86.2 | 9.3 |  | 2 | Flavonoids |
| 64 | (+)-Pinoresinolin | C_20_H_22_O_7_ | -3.87 | 374.13655 | 374.1351 | 17.072 | 0 | 0 |  |  |  | 2 | Lignans |
| 65 | Glycyroside | C_27_H_30_O_13_ | -3.5 | 562.16864 | 562.16667 | 17.272 | 0 | 0 |  |  |  | 2 | Flavonoids |
| 66 | Indican | C_14_H_17_NO_6_ | -3.9 | 295.10559 | 295.10444 | 17.306 | 0 | 0 |  |  |  | 2 | Glycoside |
| 67 | Alternariolmethylether | C_15_H_12_O_5_ | -2.49 | 272.06847 | 272.0678 | 17.337 | 5 | 0 | 77.4 | 8.9 |  | 2 |  |

**Continued Table S1 UPLC-QTOF-MS/MS Identification Results of Compounds in SFJD**

| No | identity | Molecular formula | Error（ppm） | Observed mass  (m/z) | Calculated mass  (m/z) | RT （min） | mzCloud Results | mzVault Results | mzCloud Best Match | mzCloud Best  Match Confidence | mzVault Best Match | MS Depth | Type of chemical composition |
| --- | --- | --- | --- | --- | --- | --- | --- | --- | --- | --- | --- | --- | --- |
| 68 | 4',7-Dihydroxyflavanone | C_15_H_12_O_4_ | -4.27 | 256.07356 | 256.07247 | 17.366 | 2 | 0 | 92.2 | 74.7 |  | 2 | Flavonoids |
| 69 | Indole-3-butyric acid | C_12_H_13_NO_2_ | -2.89 | 203.09463 | 203.09404 | 17.565 | 2 | 0 | 82.2 | 9.1 |  | 2 | Indoles |
| 70 | N-(p-Coumaroyl) serotonin | C_19_H_18_N_2_O_3_ | -3.12 | 322.13174 | 322.13074 | 17.633 | 1 | 0 | 82.6 | 9.1 |  | 2 | Amide |
| 71 | Licuroside | C_26_H_30_O_13_ | -3.5 | 550.16864 | 550.16672 | 17.71 | 0 | 0 |  |  |  | 2 | Flavonoids |
| 72 | Isolariciresinol | C_20_H_24_O_6_ | -4.11 | 360.15729 | 360.15581 | 17.744 | 0 | 0 |  |  |  | 2 | Lignans |
| 73 | Isoliquiritoside | C_21_H_22_O_9_ | -3.56 | 418.12638 | 418.1249 | 17.825 | 0 | 0 |  |  |  | 2 | Flavonoids |

**Continued Table S1 UPLC-QTOF-MS/MS Identification Results of Compounds in SFJD**

| No | identity | Molecular formula | Error（ppm） | Observed mass  (m/z) | Calculated mass  (m/z) | RT （min） | mzCloud Results | mzVault Results | mzCloud Best Match | mzCloud Best  Match Confidence | mzVault Best Match | MS Depth | Type of chemical composition |
| --- | --- | --- | --- | --- | --- | --- | --- | --- | --- | --- | --- | --- | --- |
| 74 | Chrysophanol | C_15_H_10_O_4_ | -3.36 | 254.05791 | 254.05705 | 18.069 | 3 | 0 | 85.2 | 9.3 |  | 2 | Anthraquinones |
| 75 | Isorhamnetin | C_16_H_12_O_7_ | -3.67 | 316.0583 | 316.05714 | 18.203 | 0 | 0 |  |  |  | 2 | Flavonoids |
| 76 | 3,4',5'-Trihydroxy-5-methoxy-2'-methyl-2-biphenylcarboxylic acid | C_15_H_14_O_6_ | -7.09 | 290.07904 | 290.07698 | 18.456 | 1 | 0 | 75.2 | 27.5 |  | 2 | Organic acid |
| 77 | 5,7-Dihydroxy-2-(4-hydroxy-3-methoxyphenyl)-6-(3-methyl-2-buten-1-yl)-2,3-dihydro-4H-chromen-4-one | C_21_H_24_O_6_ | -48399.16 | 372.15729 | 354.14519 | 18.507 | 1 | 0 | 70.1 | 58.5 |  | 2 | Flavonoids |
| 78 | Licoagrochalcone B | C_21_H_20_O_4_ | -5.09 | 336.13616 | 336.13445 | 18.508 | 0 | 0 |  |  |  | 2 | Flavonoids |

**Continued Table S1 UPLC-QTOF-MS/MS Identification Results of Compounds in SFJD**

| No | identity | Molecular formula | Error（ppm） | Observed mass  (m/z) | Calculated mass  (m/z) | RT （min） | mzCloud Results | mzVault Results | mzCloud Best Match | mzCloud Best  Match Confidence | mzVault Best Match | MS Depth | Type of chemical composition |
| --- | --- | --- | --- | --- | --- | --- | --- | --- | --- | --- | --- | --- | --- |
| 79 | Chrysin | C_15_H_10_O_4_ | -4.08 | 254.05791 | 254.05687 | 18.561 | 3 | 0 | 87.3 | 34.9 |  | 2 | Flavonoids |
| 80 | T-2 Toxin | C_24_H_34_O_9_ | 47145.14 | 466.22028 | 488.2003 | 19.362 | 1 | 0 | 82.1 | 9.1 |  | 2 |  |
| 81 | Wogonoside | C_22_H_20_O_11_ | -3.5 | 460.10056 | 460.09895 | 19.402 | 0 | 0 |  |  |  | 2 | Flavonoids |
| 82 | Kaempferol | C_15_H_10_O_6_ | -3.38 | 286.04774 | 286.04677 | 19.462 | 5 | 0 | 77.8 | 8.9 |  | 2 | Flavonoids |
| 83 | (-)-Deoxypodorhizone | C_22_H_24_O_7_ | -3.42 | 400.1522 | 400.15083 | 19.468 | 0 | 0 |  |  |  | 2 | Lignans |
| 84 | Biochanin A | C_16_H_12_O_5_ | -3.56 | 284.06847 | 284.06746 | 19.509 | 4 | 0 | 82.9 | 9.1 |  | 2 | Flavonoids |

**Continued Table S1 UPLC-QTOF-MS/MS Identification Results of Compounds in SFJD**

| No | identity | Molecular formula | Error（ppm） | Observed mass  (m/z) | Calculated mass  (m/z) | RT （min） | mzCloud Results | mzVault Results | mzCloud Best Match | mzCloud Best  Match Confidence | mzVault Best Match | MS Depth | Type of chemical composition |
| --- | --- | --- | --- | --- | --- | --- | --- | --- | --- | --- | --- | --- | --- |
| 85 | (5S,6S)-5-Hydroxy-4-methoxy-6-[(E)-2-phenylvinyl]-5,6-dihydro-2H-pyran-2-one | C_14_H_14_O_4_ | -3.43 | 246.08921 | 246.08837 | 19.549 | 6 | 0 | 81.6 | 9.1 |  | 2 |  |
| 86 | Trioxsalen | C_14_H_12_O_3_ | -3.14 | 228.07864 | 228.07793 | 19.549 | 8 | 0 | 77.4 | 8.9 |  | 2 | Coumarins |
| 87 | (6,6-Dimethylbicyclo[3.1.1]hept-2-yl)methyl 6-O-[(2R,3R,4R)-3,4-dihydroxy-4-(hydroxymethyl)tetrahydro-2-furanyl]-β-D-glucopyranoside | C_21_H_36_O_10_ | 37983.07 | 448.23085 | 465.25603 | 19.676 | 1 | 0 | 85.1 | 9.3 |  | 2 | Amide |

**Continued Table S1 UPLC-QTOF-MS/MS Identification Results of Compounds in SFJD**

| No | identity | Molecular formula | Error（ppm） | Observed mass  (m/z) | Calculated mass  (m/z) | RT （min） | mzCloud Results | mzVault Results | mzCloud Best Match | mzCloud Best  Match Confidence | mzVault Best Match | MS Depth | Type of chemical composition |
| --- | --- | --- | --- | --- | --- | --- | --- | --- | --- | --- | --- | --- | --- |
| 88 | Emodin | C_15_H_10_O_5_ | -3.43 | 270.05282 | 270.0519 | 19.832 | 8 | 0 | 83.9 | 32.8 |  | 2 | Anthraquinones |
| 89 | Matairesinol | C_20_H_22_O_6_ | -50291.97 | 358.14164 | 340.12999 | 19.974 | 1 | 0 | 69.2 | 58.2 |  | 2 | Lignans |
| 90 | Glabrene | C_20_H_18_O_4_ | -3.88 | 322.12051 | 322.11926 | 19.975 | 0 | 0 |  |  |  | 2 | Flavonoids |
| 91 | Kumatakenin | C_17_H_14_O_6_ | -3.42 | 314.07904 | 314.07796 | 19.994 | 2 | 0 | 85.8 | 9.3 |  | 2 | Flavonoids |
| 92 | Liquoric acid | C_30_H_44_O_5_ | -3.67 | 484.31887 | 484.31709 | 20.809 | 0 | 0 |  |  |  | 2 | Organic acid |
| 93 | 3,5-di-tert-Butylbenzaldehyde | C_15_H_22_O | -2.77 | 218.16707 | 218.16646 | 20.925 | 2 | 0 | 64.6 | 57 |  | 2 |  |
| 94 | Gancaonin P 3'methyl ether | C_21_H_20_O_7_ | -3.35 | 384.1209 | 384.11962 | 21.313 | 1 | 0 | 65.6 | 8.3 |  | 2 | Phenols |
| 95 | Galangin | C_15_H_10_O_5_ | -3.32 | 270.05282 | 270.05193 | 21.452 | 10 | 0 | 84.6 | 9.2 |  | 2 | Flavonoids |

**Continued Table S1 UPLC-QTOF-MS/MS Identification Results of Compounds in SFJD**

| No | identity | Molecular formula | Error（ppm） | Observed mass  (m/z) | Calculated mass  (m/z) | RT （min） | mzCloud Results | mzVault Results | mzCloud Best Match | mzCloud Best  Match Confidence | mzVault Best Match | MS Depth | Type of chemical composition |
| --- | --- | --- | --- | --- | --- | --- | --- | --- | --- | --- | --- | --- | --- |
| 96 | 12-Oxo phytodienoic acid | C_18_H_28_O_3_ | -61640.31 | 292.20384 | 274.19231 | 21.591 | 2 | 0 | 88.6 | 85.5 |  | 2 | Organic acid |
| 97 | 9S,13R-12-Oxophytodienoic acid | C_18_H_28_O_3_ | -3.87 | 292.20384 | 292.20271 | 21.591 | 3 | 0 | 87.8 | 84.3 |  | 2 | Organic acid |
| 98 | Cubebin | C_20_H_20_O_6_ | -3.84 | 356.12599 | 356.12462 | 21.723 | 0 | 0 |  |  |  | 2 | Lignans |
| 99 | Questinol | C_16_H_12_O_6_ | -3.72 | 300.06339 | 300.06227 | 21.778 | 3 | 0 | 88.8 | 9.4 |  | 2 | Anthraquinones |
| 100 | Aflatoxin G2 | C_17_H_14_O_7_ | -4.13 | 330.07395 | 330.07259 | 21.854 | 2 | 0 | 82.3 | 9.1 |  | 2 | Aflatoxin |
| 101 | 3-Hydroxy-11-oxo-12-oleanen-30,22-olide | C_30_H_44_O_4_ | -3.58 | 468.32396 | 468.32228 | 22.209 | 0 | 0 |  |  |  | 2 |  |

**Continued Table S1 UPLC-QTOF-MS/MS Identification Results of Compounds in SFJD**

| No | identity | Molecular formula | Error（ppm） | Observed mass  (m/z) | Calculated mass  (m/z) | RT （min） | mzCloud Results | mzVault Results | mzCloud Best Match | mzCloud Best  Match Confidence | mzVault Best Match | MS Depth | Type of chemical composition |
| --- | --- | --- | --- | --- | --- | --- | --- | --- | --- | --- | --- | --- | --- |
| 102 | omega-Hydroxyemodin | C_15_H_10_O_6_ | -4.03 | 286.04774 | 286.04659 | 22.383 | 4 | 0 | 76.8 | 8.8 |  | 2 | Anthraquinones |
| 103 | Ginsenoside Rb1 | C_54_H_92_O_23_ | 19823.89 | 1108.60294 | 1130.57976 | 22.513 | 1 | 0 | 87.6 | 9.4 |  | 2 | Triterpenoids |
| 104 | Pinolenic acid | C_18_H_30_O_2_ | -4 | 278.22458 | 278.22347 | 22.515 | 3 | 0 | 88 | 84.6 |  | 2 | Organic acid |
| 105 | 13(S)-HOTrE | C_18_H_30_O_3_ | -3.51 | 294.21949 | 294.21846 | 22.789 | 3 | 0 | 84 | 58.3 |  | 2 | Organic acid |
| 106 | Eicosapentaenoic acid | C_20_H_30_O_2_ | -3.68 | 302.22458 | 302.22347 | 22.939 | 12 | 0 | 86.3 | 82.1 |  | 2 | Organic acid |
| 107 | Echinatin | C_16_H_14_O_4_ | -3.46 | 270.08921 | 270.08827 | 23.211 | 1 | 0 | 64 | 38.5 |  | 2 | Flavonoids |
| 108 | 6,18,19-Trihydroxytrachyloban-2-one | C_20_H_30_O_4_ | -3.69 | 334.21441 | 334.21318 | 23.262 | 15 | 0 | 81.3 | 74.8 |  | 2 |  |

**Continued Table S1 UPLC-QTOF-MS/MS Identification Results of Compounds in SFJD**

| **No** | **identity** | **Molecular formula** | **Error（ppm）** | **Observed mass**  **(m/z)** | **Calculated mass**  **(m/z)** | **RT （min）** | **mzCloud Results** | **mzVault Results** | **mzCloud Best Match** | **mzCloud Best**  **Match Confidence** | **mzVault Best Match** | **MS Depth** | **Type of chemical composition** |
| --- | --- | --- | --- | --- | --- | --- | --- | --- | --- | --- | --- | --- | --- |
| 109 | Isoliquiritigenin | C_15_H_12_O_4_ | -3.91 | 256.07356 | 256.07256 | 23.395 | 2 | 0 | 92.9 | 77.5 |  | 2 | Flavonoids |
| 110 | Scoparone | C_11_H_10_O_4_ | -2.89 | 206.05791 | 206.05731 | 23.473 | 2 | 0 | 81.7 | 9.1 |  | 2 | Coumarins |
| 111 | Formononetin | C_16_H_12_O_4_ | -3.87 | 268.07356 | 268.07252 | 23.679 | 4 | 0 | 92.9 | 9.6 |  | 2 | Flavonoids |
| 112 | Arctigenin | C_21_H_24_O_6_ | -3.71 | 372.15729 | 372.15591 | 23.798 | 0 | 0 |  |  |  | 2 | Lignans |
| 113 | 4-[(1S,3aR,4S,6aR)-4-(3,4-Dimethoxyphenyl)tetrahydro-1H,3H-furo[3,4-c]furan-1-yl]-2-methoxyphenol | C_21_H_24_O_6_ | -48398.67 | 372.15729 | 354.14537 | 23.805 | 1 | 0 | 88 | 84.6 |  | 2 |  |
| 114 | Afrormosin | C_17_H_14_O_5_ | -3.48 | 298.08412 | 298.08309 | 24.17 | 0 | 0 |  |  |  | 2 | Flavonoids |

**Continued Table S1 UPLC-QTOF-MS/MS Identification Results of Compounds in SFJD**

| No | identity | Molecular formula | Error（ppm） | Observed mass  (m/z) | Calculated mass  (m/z) | RT （min） | mzCloud Results | mzVault Results | mzCloud Best Match | mzCloud Best  Match Confidence | mzVault Best Match | MS Depth | Type of chemical composition |
| --- | --- | --- | --- | --- | --- | --- | --- | --- | --- | --- | --- | --- | --- |
| 115 | 18beta-glycyrrhetinic acid | C_30_H_46_O_4_ | -3.88 | 470.33961 | 470.33779 | 24.694 | 2 | 0 | 91.8 | 73.2 |  | 2 | Triterpenoids |
| 116 | Chinensinaphthol | C_21_H_16_O_7_ | -3.92 | 380.0896 | 380.08811 | 24.761 | 0 | 0 |  |  |  | 2 | Lignans |
| 117 | Tricin | C_17_H_14_O_7_ | -3.95 | 330.07395 | 330.07265 | 25.147 | 1 | 0 | 84.5 | 9.2 |  | 2 | Flavonoids |
| 118 | Icaritin | C_21_H_20_O_6_ | -3.76 | 368.12599 | 368.1246 | 25.153 | 1 | 0 | 73.6 | 44.5 |  | 2 | Flavonoids |
| 119 | 1-O-{(3β,5ξ,9ξ,18ξ)-3-[(3-O-Hexopyranosylhexopyranuronosyl)oxy]-28-oxoolean-12-en-28-yl}hexopyranose | C_48_H_76_O_19_ | -4.19 | 956.49808 | 956.49407 | 25.448 | 1 | 0 | 82.1 | 31.7 |  | 2 |  |

**Continued Table S1 UPLC-QTOF-MS/MS Identification Results of Compounds in SFJD**

| No | identity | Molecular formula | Error（ppm） | Observed mass  (m/z) | Calculated mass  (m/z) | RT （min） | mzCloud Results | mzVault Results | mzCloud Best Match | mzCloud Best  Match Confidence | mzVault Best Match | MS Depth | Type of chemical composition |
| --- | --- | --- | --- | --- | --- | --- | --- | --- | --- | --- | --- | --- | --- |
| 120 | Oleanolic acid | C_30_H_48_O_3_ | -39469.68 | 456.36035 | 438.34795 | 25.523 | 1 | 0 | 73.8 | 44.8 |  | 2 | Triterpenoids |
| 121 | Hispidulin | C_16_H_12_O_6_ | -4.24 | 300.06339 | 300.06212 | 25.861 | 2 | 0 | 63.9 | 8.2 |  | 2 | Flavonoids |
| 122 | Glycitein | C_16_H_12_O_5_ | -3.89 | 284.06847 | 284.06737 | 25.885 | 7 | 0 | 90.2 | 9.5 |  | 2 | Flavonoids |
| 123 | Ursolic acid lactone | C_30_H_46_O_3_ | -3.65 | 454.3447 | 454.34303 | 25.925 | 0 | 0 |  |  |  | 2 | Triterpenoids |
| 124 | Licoleafol | C_20_H_20_O_7_ | -3.71 | 372.1209 | 372.11952 | 26.446 | 0 | 0 |  |  |  | 2 | Flavonoids |
| 125 | 5-OxoETE | C_20_H_30_O_3_ | -69082.21 | 318.21949 | 296.23619 | 26.556 | 6 | 0 | 81.2 | 74.7 |  | 2 |  |
| 126 | 3'-Methoxyglabridin | C_21_H_22_O_5_ | -3.64 | 354.14672 | 354.14543 | 26.62 | 0 | 0 |  |  |  | 2 |  |
| 127 | Shogaol | C_17_H_24_O_3_ | -3.8 | 276.17254 | 276.1715 | 26.645 | 2 | 0 | 86.3 | 9.3 |  | 2 | Phenols |

**Continued Table S1 UPLC-QTOF-MS/MS Identification Results of Compounds in SFJD**

| No | identity | Molecular formula | Error（ppm） | Observed mass  (m/z) | Calculated mass  (m/z) | RT （min） | mzCloud Results | mzVault Results | mzCloud Best Match | mzCloud Best  Match Confidence | mzVault Best Match | MS Depth | Type of chemical composition |
| --- | --- | --- | --- | --- | --- | --- | --- | --- | --- | --- | --- | --- | --- |
| 128 | Methyl palmitate | C_17_H_34_O_2_ | 62997.06 | 270.25588 | 287.28121 | 26.824 | 2 | 0 | 90.8 | 38.7 |  | 2 | Miscellaneous |
| 129 | Indirubin | C_16_H_10_N_2_O_2_ | -3.69 | 262.07423 | 262.07326 | 26.97 | 1 | 0 | 77.8 | 8.9 |  | 2 | Alkaloids |
| 130 | Kaerophyllin | C_21_H_20_O_6_ | -3.8 | 368.12599 | 368.12459 | 27.539 | 2 | 0 | 76 | 8.8 |  | 2 | Lignans |
| 131 | Gancaonin F | C_21_H_16_O_6_ | -3.51 | 364.09469 | 364.09341 | 27.686 | 0 | 0 |  |  |  | 2 | Phenols |
| 132 | Glyasperin C | C_21_H_24_O_5_ | -3.86 | 356.16237 | 356.161 | 27.917 | 0 | 0 |  |  |  | 2 | Flavonoids |
| 133 | Aflatoxin B2 | C_17_H_14_O_6_ | -3.82 | 314.07904 | 314.07784 | 27.93 | 2 | 0 | 89.2 | 9.5 |  | 2 | Aflatoxin |
| 134 | Wighteone | C_20_H_18_O_5_ | -4.03 | 338.11542 | 338.11406 | 27.935 | 0 | 0 |  |  |  | 2 | Flavonoids |
| 135 | Isochaihulactone | C_22_H_22_O_7_ | -3.83 | 398.13655 | 398.13503 | 28.722 | 0 | 0 |  |  |  | 2 | Coumarins |

**Continued Table S1 UPLC-QTOF-MS/MS Identification Results of Compounds in SFJD**

| No | identity | Molecular formula | Error（ppm） | Observed mass  (m/z) | Calculated mass  (m/z) | RT （min） | mzCloud Results | mzVault Results | mzCloud Best Match | mzCloud Best  Match Confidence | mzVault Best Match | MS Depth | Type of chemical composition |
| --- | --- | --- | --- | --- | --- | --- | --- | --- | --- | --- | --- | --- | --- |
| 136 | 2-Aminooctadec-4-yne-1,3-diol | C_18_H_35_NO_2_ | -3.83 | 297.26678 | 297.26564 | 28.859 | 1 | 0 | 82 | 75.8 |  | 2 |  |
| 137 | Isotrifoliol | C_16_H_10_O_6_ | -4.07 | 298.04774 | 298.04653 | 29.158 | 0 | 0 |  |  |  | 2 | Flavonoids |
| 138 | Laburnetin | C_20_H_18_O_6_ | -3.62 | 354.11034 | 354.10906 | 29.159 | 0 | 0 |  |  |  | 2 |  |
| 139 | Licochalcone A | C_21_H_22_O_4_ | -3.69 | 338.15181 | 338.15056 | 29.188 | 1 | 0 | 65 | 57.1 |  | 2 | Flavonoids |
| 140 | Licoricone | C_22_H_22_O_6_ | -3.73 | 382.14164 | 382.14021 | 29.23 | 0 | 0 |  |  |  | 2 | Flavonoids |
| 141 | Licochalcone C | C_21_H_22_O_4_ | -3.69 | 338.15181 | 338.15056 | 29.508 | 2 | 0 | 87.9 | 84.5 |  | 2 | Flavonoids |
| 142 | Bis(4-ethylbenzylidene)sorbitol | C_24_H_30_O_6_ | -3.97 | 414.20424 | 414.20259 | 29.646 | 1 | 0 | 89.2 | 9.5 |  | 2 | Saccharides |
| 143 | Naringenin | C_15_H_12_O_5_ | -3.38 | 272.06847 | 272.06755 | 29.917 | 5 | 0 | 83.4 | 9.2 |  | 2 | Flavonoids |

**Continued Table S1 UPLC-QTOF-MS/MS Identification Results of Compounds in SFJD**

| No | identity | Molecular formula | Error（ppm） | Observed mass  (m/z) | Calculated mass  (m/z) | RT （min） | mzCloud Results | mzVault Results | mzCloud Best Match | mzCloud Best  Match Confidence | mzVault Best Match | MS Depth | Type of chemical composition |
| --- | --- | --- | --- | --- | --- | --- | --- | --- | --- | --- | --- | --- | --- |
| 144 | Glycyrol | C_21_H_18_O_6_ | -3.92 | 366.11034 | 366.1089 | 30.144 | 0 | 0 |  |  |  | 2 | Coumarins |
| 145 | Glabrone | C_20_H_16_O_5_ | -3.71 | 336.09977 | 336.09853 | 30.496 | 2 | 0 | 67.7 | 8.4 |  | 2 | Flavonoids |
| 146 | Isobavachalcone | C_20_H_20_O_4_ | -3.78 | 324.13616 | 324.13494 | 30.624 | 2 | 0 | 90.1 | 66.6 |  | 2 | Flavonoids |
| 147 | Isomucronulatol | C_17_H_18_O_5_ | -3.7 | 302.11542 | 302.11431 | 30.863 | 0 | 0 |  |  |  | 2 | Flavonoids |
| 148 | Abietic acid | C_20_H_30_O_2_ | -3.58 | 302.22458 | 302.2235 | 31.054 | 20 | 0 | 84.6 | 79.7 |  | 2 | Triterpenoids |
| 149 | Gancaonin A | C_21_H_20_O_5_ | -3.68 | 352.13107 | 352.12978 | 31.206 | 0 | 0 |  |  |  | 2 | Flavonoids |
| 150 | Calocarpin | C_20_H_20_O_4_ | -3.78 | 324.13616 | 324.13494 | 31.408 | 1 | 0 | 76 | 67.2 |  | 2 |  |
| 151 | Kanzonol T | C_25_H_26_O_7_ | -3.25 | 438.16785 | 438.16643 | 31.408 | 0 | 0 |  |  |  | 2 | Flavonoids |
| 152 | Licoisoflavone B | C_20_H_16_O_6_ | -3.92 | 352.09469 | 352.09331 | 31.671 | 0 | 0 |  |  |  | 2 | Flavonoids |

**Continued Table S1 UPLC-QTOF-MS/MS Identification Results of Compounds in SFJD**

| **No** | **identity** | **Molecular formula** | **Error（ppm）** | **Observed mass**  **(m/z)** | **Calculated mass**  **(m/z)** | **RT （min）** | **mzCloud Results** | **mzVault Results** | **mzCloud Best Match** | **mzCloud Best**  **Match Confidence** | **mzVault Best Match** | **MS Depth** | **Type of chemical composition** |
| --- | --- | --- | --- | --- | --- | --- | --- | --- | --- | --- | --- | --- | --- |
| 153 | 9-(Acetyloxy)-8,8-dimethyl-2-oxo-9,10-dihydro-2H,8H-pyrano[2,3-f]chromen-10-yl 2-methyl-2-butenoate | C_21_H_22_O_7_ | 44090.76 | 386.13655 | 403.16161 | 32.379 | 1 | 0 | 89.4 | 9.5 |  | 2 |  |
| 154 | Homobutein | C_16_H_14_O_5_ | -4.16 | 286.08412 | 286.08293 | 32.38 | 0 | 0 |  |  |  | 2 | Flavonoids |
| 155 | 1-Linoleoyl glycerol | C_21_H_38_O_4_ | -50842.03 | 354.27701 | 336.26485 | 32.384 | 2 | 0 | 89 | 35.9 |  | 2 |  |
| 156 | (+)-Nootkatone | C_15_H_22_O | -3.05 | 218.16707 | 218.1664 | 32.536 | 1 | 0 | 71.5 | 25.3 |  | 2 | Triterpenoids |
| 157 | 7,4'-Dihydroxy-3'-methoxyisoflavan | C_16_H_16_O_4_ | -3.64 | 272.10486 | 272.10387 | 32.969 | 0 | 0 |  |  |  | 2 | Coumarins |

**Continued Table S1 UPLC-QTOF-MS/MS Identification Results of Compounds in SFJD**

| No | identity | Molecular formula | Error（ppm） | Observed mass  (m/z) | Calculated mass  (m/z) | RT （min） | mzCloud Results | mzVault Results | mzCloud Best Match | mzCloud Best  Match Confidence | mzVault Best Match | MS Depth | Type of chemical composition |
| --- | --- | --- | --- | --- | --- | --- | --- | --- | --- | --- | --- | --- | --- |
| 158 | Kanzonol R | C_22_H_26_O_5_ | -3.62 | 370.17802 | 370.17668 | 33.222 | 0 | 0 |  |  |  | 2 | Flavonoids |
| 159 | Psoralidin | C_20_H_16_O_5_ | -4.25 | 336.09977 | 336.09834 | 33.294 | 6 | 0 | 76.5 | 8.8 |  | 2 | Coumarins |
| 160 | Linolenic acid | C_18_H_30_O_2_ | -3.34 | 278.22458 | 278.22365 | 34.268 | 6 | 0 | 83.3 | 32.5 |  | 2 | Miscellaneous |
| 161 | Macarangaflavanone B | C_25_H_28_O_5_ | -3.35 | 408.19367 | 408.19231 | 35.038 | 0 | 0 |  |  |  | 2 | Flavonoids |
| 162 | 5,7,3',4'-Tetrahydroxy-6,8-diprenylisoflavone | C_25_H_26_O_6_ | -3.85 | 422.17294 | 422.17131 | 35.135 | 1 | 0 | 67.3 | 8.4 |  | 2 | Flavonoids |
| 163 | Senegalensin | C_25_H_26_O_6_ | -3.85 | 422.17294 | 422.17131 | 35.467 | 1 | 0 | 77 | 8.8 |  | 2 | Flavonoids |

**Continued Table S1 UPLC-QTOF-MS/MS Identification Results of Compounds in SFJD**

| No | identity | Molecular formula | Error（ppm） | Observed mass  (m/z) | Calculated mass  (m/z) | RT （min） | mzCloud Results | mzVault Results | mzCloud Best Match | mzCloud Best  Match Confidence | mzVault Best Match | MS Depth | Type of chemical composition |
| --- | --- | --- | --- | --- | --- | --- | --- | --- | --- | --- | --- | --- | --- |
| 164 | 9-Oxo-10(E),12(E)-octadecadienoic acid | C_18_H_30_O_3_ | -4.24 | 294.21949 | 294.21825 | 36.133 | 9 | 0 | 92.3 | 92.3 |  | 2 |  |
| 165 | Dibutyl phthalate | C_16_H_22_O_4_ | -3.09 | 278.15181 | 278.15095 | 36.617 | 6 | 0 | 81.6 | 9.1 |  | 2 | Lipid |
| 166 | Citroflex 4 | C_18_H_32_O_7_ | -4.31 | 360.2148 | 360.21325 | 36.663 | 1 | 0 | 94.7 | 9.7 |  | 2 | Lipid |
| 167 | erybacin B | C_19_H_18_O_5_ | -3.99 | 326.11542 | 326.11412 | 36.828 | 0 | 0 |  |  |  | 2 |  |
| 168 | Gancaonin Q | C_25_H_26_O_5_ | -3.9 | 406.17802 | 406.17644 | 37.452 | 0 | 0 |  |  |  | 2 | Flavonoids |
| 169 | (+/-)11(12)-EET | C_20_H_32_O_3_ | -4.11 | 320.23514 | 320.23383 | 41.274 | 12 | 0 | 85.9 | 60.9 |  | 2 |  |
| 170 | Erucamide | C_22_H_43_NO | -4.18 | 337.33446 | 337.33306 | 46.872 | 2 | 0 | 87.7 | 63.3 |  | 2 | Amide |

**Continued Table S1 UPLC-QTOF-MS/MS Identification Results of Compounds in SFJD**

| **No** | **identity** | **Molecular formula** | **Error（ppm）** | **Observed mass**  **(m/z)** | **Calculated mass**  **(m/z)** | **RT （min）** | **mzCloud Results** | **mzVault Results** | **mzCloud Best Match** | **mzCloud Best**  **Match Confidence** | **mzVault Best Match** | **MS Depth** | **Type of chemical composition** |
| --- | --- | --- | --- | --- | --- | --- | --- | --- | --- | --- | --- | --- | --- |
| 171 | Stearamide | C_18_H_37_NO | -3.63 | 283.28751 | 283.28649 | 47.508 | 2 | 0 | 86.1 | 9.3 |  | 2 | Amide |
